# Multiplicity of acquired cross-resistance in paclitaxel-resistant cancer cells is associated with feedback control of TUBB3 via FOXO3a-mediated ABCB1 regulation

**DOI:** 10.18632/oncotarget.9118

**Published:** 2016-04-30

**Authors:** Mark Borris D. Aldonza, Ji-Young Hong, Malona V. Alinsug, Jayoung Song, Sang Kook Lee

**Affiliations:** ^1^ College of Pharmacy, Seoul National University, Seoul 151-742, Korea; ^2^ Department of Biochemistry, College of Veterinary Medicine, Seoul National University, Seoul 151-742, Korea; ^3^ Center for Food and Bioconvergence, College of Agriculture and Life Sciences, Seoul National University, Seoul 151-742, Korea; ^4^ Present address: Department of Biological Sciences, Korea Advanced Institute of Science and Technology (KAIST), Daejeon 305-701, Korea

**Keywords:** paclitaxel resistance, multidrug resistance, FOXO3a, TUBB3, ABCB1

## Abstract

Acquired drug resistance is a primary obstacle for effective cancer therapy. The correlation of point mutations in class III β-tubulin (*TUBB3*) and the prominent overexpression of ATP-binding cassette P-glycoprotein (*ABCB1*), a multidrug resistance gene, have been protruding mechanisms of resistance to microtubule disruptors such as paclitaxel (PTX) for many cancers. However, the precise underlying mechanism of the rapid onset of cross-resistance to an array of structurally and functionally unrelated drugs in PTX-resistant cancers has been poorly understood. We determined that our established PTX-resistant cancer cells display *ABCB1/ABCC1*-associated cross-resistance to chemically different drugs such as 5-fluorouracil, docetaxel, and cisplatin. We found that feedback activation of *TUBB3* can be triggered through the *FOXO3a*-dependent regulation of *ABCB1*, which resulted in the accentuation of induced PTX resistance and encouraged multiplicity in acquired cross-resistance. *FOXO3a*-directed regulation of P-glycoprotein (P-gp) function suggests that control of *ABCB1* involves methylation-dependent activation. Consistently, transcriptional overexpression or downregulation of *FOXO3a* directs inhibitor-controlled protease-degradation of TUBB3. The functional PI3K/Akt signaling is tightly responsive to *FOXO3a* activation alongside doxorubicin treatment, which directs *FOXO3a* arginine hypermethylation. In addition, we found that secretome factors from PTX-resistant cancer cells with acquired cross-resistance support a P-gp-dependent association in multidrug resistance (MDR) development, which assisted the *FOXO3a*-mediated control of *TUBB3* feedback. The direct silencing of *TUBB3* reverses induced multiple cross-resistance, reduces drug-resistant tumor mass, and suppresses the impaired microtubule stability status of PTX-resistant cells with transient cross-resistance. These findings highlight the control of the *TUBB3* response to *ABCB1* genetic suppressors as a mechanism to reverse the profuse development of multidrug resistance in cancer.

## INTRODUCTION

Multidrug resistance (MDR) in cancer is a phenomenal limitation to the success of chemotherapy in which cancer cells gain the capacity to develop cross-resistance to a broad range of structurally and functionally unrelated drugs. Among the well-known mechanisms underlying MDR is the hyperactivity of drug efflux by ATP-binding cassette (ABC) transporters bound in the cell membrane compartments. The overexpression of ABC transcription correlates with an increase in patient relapse during or following treatment with various chemotherapy drugs [1, 2]. Paclitaxel (PTX), an anticancer drug against various human solid tumors, primarily targets microtubules (mt) to disrupt mitosis, motility, and the growth of cells. PTX-resistant cancers highlight the rapid onset of multiple cross-resistance and the high percentage of failures even in therapies that involve drug combinations. Alterations in tubulin-isoform expression alongside the overexpression of energy-driven P-glycoprotein (P-gp; encoded by *ABCB1*) are well-known causative factors of taxane-refractory tumors. Mt-targeting agents such as PTX and its synthetic analogue docetaxel (DCT) bind to the β-subunit in the αβ-tubulin dimer, which exemplifies the stability of the mt assembly [3, 4]. In several cancers, class III β-tubulin (encoded by *TUBB3*) is a predictive biomarker of clinical PTX resistance and a DCT-based chemotherapy response [5]. A number of factors are involved in the concession of mt-targeted therapies including the patient's β-tubulin isotype differential expression profile in various tissues/organs and the modifications in tubulin-binding sites, mt assembly properties, tubulin synthesis, and tubulin polymerization. Dynamic equilibrium between soluble tubulin dimers are maintained by microtubule-associated proteins (MAPs) collectively known as microtubule inner proteins (MIPs), which include proteins that are intact with tubulin heterodimers. This specific occasion of equipoise is often targeted by PTX but is altered in several malignancies [6, 7]. Conflicting results and inconsistencies have previously been identified in both *in vitro* and *in vivo* models of the mechanism of MAPs in drug resistance, which suggests that there is much work remaining to elucidate the precise mechanisms of action.

A rapidly growing paradigm is that targeted therapies require factors that can overcome the spontaneous mutations in β-tubulin isotypes to reverse resistance to PTX and other taxanes [8]. Therefore, designing small molecule drugs and testing rationale drug combinations that can target specific β-tubulin isotype modifications to reverse P-gp-mediated resistance are warranted; however, this is very challenging because structurally, the seven isotypes of β-tubulin have complex differential functional mechanisms on mt and play key roles in cellular homeostasis [9]. Therefore, the discovery of genes that can regulate the feedback control of β-tubulin isotypes associated with drug sensitivity is necessary to provide a rationale platform for both MDR biomarkers and therapeutic discoveries.

Forkhead box class O (FOXO) transcription factors such as *FOXO3a* have recently been identified as key players in the initiation of cancer and the development of drug resistance. The anticancer drug-mediated up-regulation of *FOXO3a* enhances *ABCB1* expression, which may directly contribute to the genesis of MDR in general and to the implicated *FOXO* activation-mediated chemotherapy response, including those cytostatic and cytotoxic effects amended by PTX, DCT, cisplatin (CIS), gefitinib (GEF), and 5-fluorouracil (5-FU) [10, 11]. Identified as downstream targets of the PI3K/Akt pathway, *FOXO* transcription factors are associated with tumorigenesis and chemotherapeutic resistance in several ways, such as through inhibiting the transactivation of drug-target genes (e.g., p27/Kip1, Bcl-xL, cyclin D, and Bim) involved in cell proliferation, apoptosis, and differentiation [12]. In addition, because the overexpression of Akt can increase resistance to PTX, FOXO transcription factors have since been implicated in determining drug sensitivity and affecting other signal transduction pathways that regulate the response to PTX. Similarly, the MAPK member JNK, specifically its sub-members JNK1 and JNK2, augment protection from the toxic effects of PTX [13, 14]. Furthermore, PTX not only induces FOXO3a expression but also enhances its nuclear translocation through a JNK-dependent mechanism and affects its ubiquitin-mediated degradation. Meanwhile, *FOXO3a*, aside from affecting PTX actions, regulates the DNA damage response and stimulates the DNA repair pathway, which regulates the sensitivity to drugs that target the DNA repair system such as 5-FU and CIS, among others [15, 16]. Forkhead box M1 (*FOXM1*) and *FOXO3a* have been observed to compete in binding to similar DNA sequences, which often results in antagonized transcriptional output that has recently been related to genotoxic drug resistance and the response of various cancers to chemotherapy [17, 18].

Considerable progress has been made in determining the mechanism of FOXO-regulated mt organization. Very recently, FOXO has also been implicated in drug-mediated cytoskeletal stress because of its effects on neuronal mt organization following pharmacological damage, which requires Akt kinase [19, 20]. Importantly, some FOXO transcription factors also influence the PTX-induced inhibition of the androgen receptor (AR), suggesting a connection between the mt-dependent trafficking of the AR and the clinical efficacy of PTX as well as that of other taxanes [21]. Although these distinct drug-induced mt organization regulatory events may suggest a connection between β-tubulin isotypes such as *TUBB3* and FOXO transcription factors, very little is known about the systemic relation of these factors and their collective function as interacting elements in the regulation of the response of cancers to chemotherapeutic drugs and the malignant progression of tumors caused by MDR that often leads to cancer recurrence.

Herein, in light of the increasing demand to uncover drug resistance mechanisms, we dissected the function of *FOXO3a*-mediated *ABCB1* in regulating *TUBB3* feedback in the context of the development of multiple cross-resistance to chemically unrelated cancer chemotherapeutics in PTX-resistant cancer cells, and we extended this event to systemic drug-resistant tumor progression.

## RESULTS

### *ABCB1*-associated acquired drug resistance is correlated with *TUBB3* and *FOXO3a* expression

**Figure 1:**
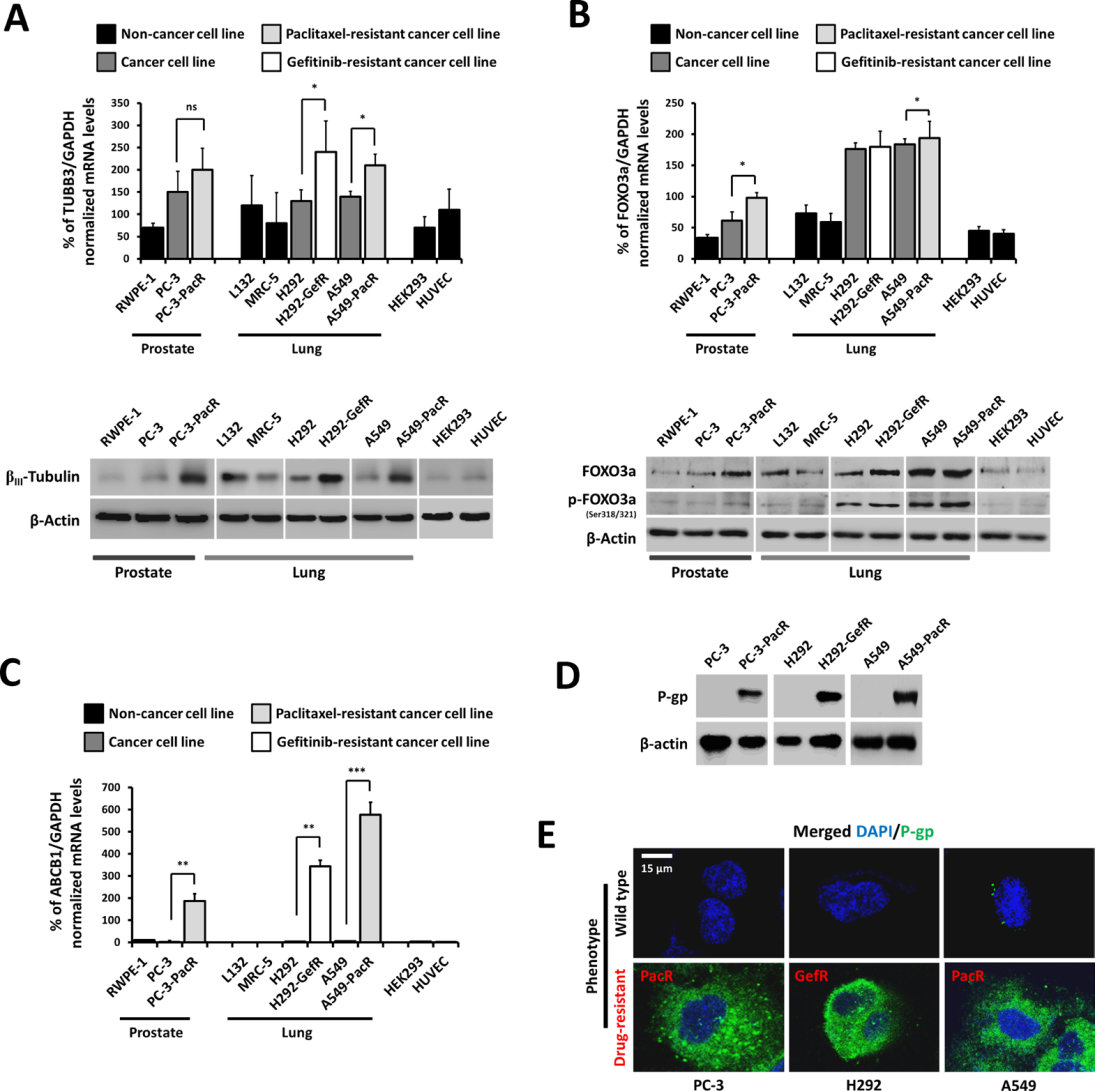
Overexpression profiles of TUBB3 and FOXO3a in a panel of cancer cells with ABCB1-associated acquired drug resistance (**A**) Characterization of indicated parental or drug-resistant phenotype cell lines for TUBB3 expression at both mRNA (upper panel) and protein (lower panel) levels. (**B**) Characterization of indicated parental or drug-resistant phenotype cell lines for FOXO3a expression at both mRNA (upper panel) and protein (lower panel) levels. (**C**) Identification of *ABCB1*-association expression in a panel of indicated parental or drug resistant cancer cell lines at the mRNA level. (**D**) Confirmation of P-gp protein overexpression in drug resistant cancer cell lines. (**E**) Intracellular distribution and localization of P-gp expression in both wild-type and drug-resistant phenotype cell lines. Cells were stained with human P-gp antibody and DAPI and analyzed through confocal microscopy. Images shown were magnified at ×200.

Given the previous reports that separately associate drug-induced FOXO3a phosphorylation and *TUBB3* alterations with the overexpression of *ABCB1* [5, 11], we sought to examine the transcription and protein expression patterns of *TUBB3* and *FOXO3a* in a panel of non-tumor (normal cell), drug-sensitive cancer, and drug-resistant cancer cell models to correlate their expression with MDR development. A gene expression analysis showed that both *TUBB3* and *FOXO3a* mRNA levels are relatively lower in non-cancer RWPE-1 prostate cells, L132 and MRC-5 lung cells, and HEK293 and HUVEC cells whereas their levels are higher in drug-sensitive cancer PC-3 prostate cells and H292 and A549 lung cells. The expressions of both *TUBB3* and *FOXO3a* were the highest in cancer cells with derived PTX and GEF resistance. In PacR cancer cells, TUBB3 expression had > 2.8 and > 1.3 fold increases while FOXO3a had > 2.8 and > 1.0 fold increases when compared to both drug-sensitive normal and cancer cells, respectively. A Western blot analysis also exhibited the same expression patterns of both genes including FOXO3a phosphorylation (Figure 1A, 1B), which showed relatively higher expressions in drug-resistant variants. Furthermore, the level of *ABCB1* gene expression was confirmed to be several-fold higher in drug-resistant cancer cells compared to normal and parental cells, which confirms the high level of drug resistance in these cells (Figure 1C). Higher P-gp expression in drug-resistant cancer cells compared to their parent cells was clearly demonstrated both in protein expression (Figure 1D) and intracellular distribution (Figure 1E).

### PTX-resistant cancer cells display frequent cross-resistance to DCT, 5-FU, and CIS associated with high P-gp-mediated drug efflux

**Figure 2:**
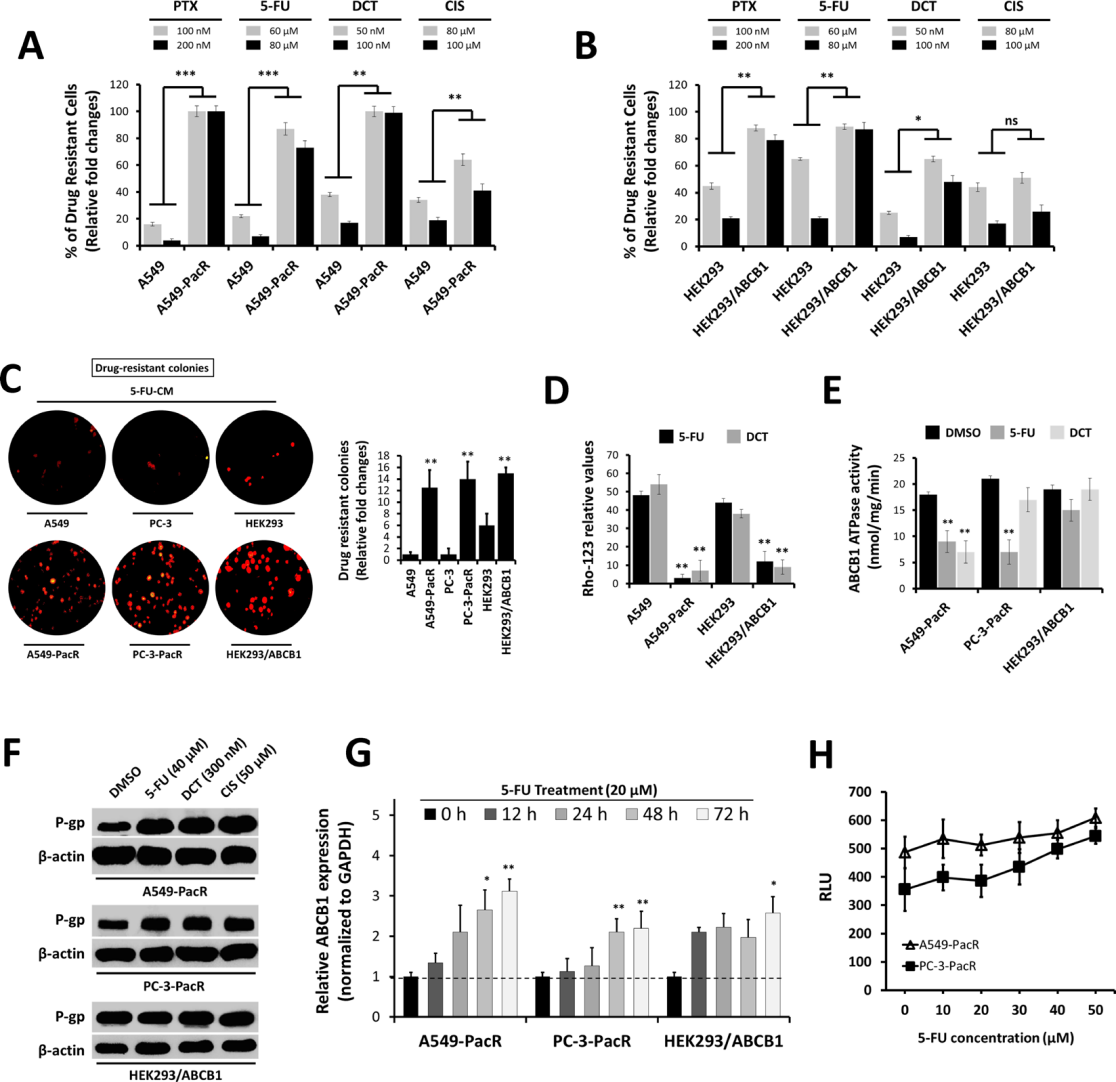
Occurrence of cross-resistance in PTX-resistant cancer cells is highly associated with ATP-dependent P-gp/ABCB1 efflux activity (**A** and **B**) Fraction of drug-intolerant A549, A549-PacR (A) and HEK293, HEK293/*ABCB1* (B) cells. Cells were treated with PTX, 5-FU, DCT, or CIS for 24 hr, and the cell viability was determined by MTT assay. Data are represented as means ± SEM. (**C**) Representative drug intolerant cell colonies (right) and quantified colony numbers of A549-PacR cells (left). Cells were exposed to 5-FU-conditioned media (CM) and continuously grown for > 7 days and formed colonies of established PTX-resistant cells were stained with sapphire 700. Data are represented as means ± SEM. (**D** and **E**) ATP-dependent P-gp efflux activity. Cells were treated with 60 μM 5-FU for 24 hr and assayed for Rho-123 incorporation. Flow cytometry was used to quantify Rho-123 fluorescence (D). Drug-resistant cells were treated with DMSO, 60 μM 5-FU, or 50 μM DCT for 24 hr and was assayed for *ABCB1* ATPase activity (E). Data are represented as means ± SEM. (**F** and **G**) Association of P-gp expression with occurrence of cross-resistance. Drug-resistant cells were treated with 5-FU, DCT, or CIS for 24 hr (F) and time-dependently treated with 5-FU for (G). Cells were treated with 20 μM 5-FU then assayed for qRT-PCR using *ABCB1*-specific primer. (**H**) P-gp-specific ATPase activity. Cells were treated with increasing 5-FU concentration for 24 hr and cells were subjected to P-gp luminescent ATPase assay. Data are represented as means ± SEM.

Several paclitaxel-resistant cancers have been defined to have cross-resistance to a variety of chemically unrelated drugs such as cisplatin, vinblastine, and anthracyclines [22, 23]. Along with the overexpression of P-gp (*ABCB1*), progress in determining other key molecular events that lead to the onset of multiple cross-resistance in PTX-resistant cancers has been greatly hampered [24]. To investigate the degree and mechanism of cross-resistance in PTX-resistant cancer cells, we employed our previously established PTX-resistant sublines, A549-PacR and PC-3-PacR cells, and developed transient cross-resistance to 5-FU, DCT, and CIS (see the Experimental Procedures). First, we identified whether P-gp drug efflux is involved in the occurrence of cross-resistance to 5-FU, DCT, and CIS in PTX-resistant cancer cells. Notably, fractions of the drug intolerant cells following treatment with apoptosis-inducing concentrations of the drugs were increased in PTX-resistant cancer cells and *ABCB1*-transfected HEK293 cells, suggesting that P-gp expression is highly correlated, except to CIS cross-resistance (Figure 2A, 2B). The number of drug-resistant colonies following exposure to 5-FU-conditioned media (CM) complemented these findings (Figure 2C). A549-PacR and HEK293/*ABCB1* cells showed very low intracellular accumulation of Rho-123 dye relative to parental and empty vector (EV)-transfected cells (Figure 2D). Paclitaxel resistant (PacR) cells displayed lower ABCB1 ATPase activity following DCT and 5-FU treatment compared to DMSO (vehicle)-treated control cells, whereas HEK293/*ABCB1* cells induced minimal lowered levels (Figure 2E). Treatment with 5-FU, DCT, and CIS positively stimulated P-gp protein expression in A549-PacR and PC-3-PacR cells (Figure 2F). Moreover, a lower 5-FU concentration regulated *ABCB1* gene expression in A549-PacR and PC-3-PacR cells in a strict time-dependent manner whereas variations in *ABCB1* gene expression were observed in HEK293/*ABCB1* cells (Figure 2G). Drug-stimulated P-gp-specific luminescent ATPase activity of both A549-PacR and PC-3-PacR cells was stimulated in a concentration-dependent manner following 5-FU treatment (Figure 2H). The cells were then screened for their response to a variety of chemotherapeutics. Both A549-PacR and PC-3-PacR cells were found to have frequent cross-resistance to DCT, 5-FU, and CIS whereas H292-GefR cells had minimal-fold cross-resistance, as determined by the IC_50_ values of incorporated drugs (Table 1). In addition, PacR cancer cells with developed transient cross-resistance showed increased resistance relative to both the parental and PacR cells in all of the drug cases tested. Specifically, PacR cancer cells with transient 5-FU cross-resistance (-PacR/5-FU) showed the highest relative resistance and P-gp dependence among others with distinct MRP (*ABCC1*) overexpression in the PC-3 subline, as determined by Western blot analysis. *ABCB1*-transfected HEK293 cells also displayed cross-resistance to 5-FU and DCT but not to CIS. These results suggest the involvement of P-gp activity in multiple cross-resistance in PTX-resistant cancer cells.

**Table 1:** Cross-drug resistance profile of cells with resistance to paclitaxel or gefitinib, and of ABCB1 transiently transfected cells and association with P-gp and MRP protein expressions

Phenotype Description	Cell Line ID	WB Grade(Protein)		5-Fluorouracil (5-FU)			Docetaxel (DCT)			Cisplatin (CIS)		
		P-gp/ABCB1	MRP/ABCC1	IC_50_ (μM) ± SD	*^a^*RR	*^b^*RR	IC_50_ (nM) ± SD	*^a^*RR	*^b^*RR	IC_50_ (μM) ± SD	*^a^*RR	*^b^*RR
Parental	A549	NE	NE	29.9 ± 0.7	ND	ND	39.8 ± 0.1	ND	ND	59.8 ± 0.8	ND	ND
Paclitaxel resistance	A549-PacR	†††	†	92.8 ± 2.1	3.10	1.00	90.8 ± 4.6	2.28	1.00	82.3 ± 4.3	1.37	1.00
Developed cross-resistance	A549-PacR/5-FU	†††	††	154.6 ± 1.0	5.17	1.66	103.2 ± 1.8	2.59	1.13	90.8 ± 4.6	1.51	1.10
	A549-PacR/DCT	†††	†	99.4 ± 3.3	3.32	1.07	102.4 ± 2.9	2.57	1.12	101.2 ± 8.1	1.69	1.22
	A549-PacR/CIS	††	†	94.5 ± 2.0	3.16	1.01	89.8 ± 2.0	2.25	0.98	122.6 ± 6.0	2.03	1.47
Parental	PC-3	NE	NE	33.7 ± 4.0	ND	ND	63.2 ± 5.9	ND	ND	29.2 ± 1.8	ND	ND
Paclitaxel resistance	PC-3-PacR	††	††	95.5 ± 5.4	2.83	1.00	97.5 ± 10.1	1.54	1.00	80.1 ± 12.4	2.74	1.00
Developed cross-resistance	PC-3-PacR/5-FU	†††	†††	142.4 ± 3.1	4.22	1.49	98.4 ± 3.1	1.55	1.01	91.2 ± 2.3	3.12	1.01
	PC-3-PacR/DCT	††	†	98.8 ± 2.1	2.93	1.03	129.2 ± 3.0	2.04	1.32	91.1 ± 10.3	3.11	1.01
	PC-3-PacR/CIS	††	NE	97.6 ± 2.7	2.89	1.02	100.2 ± 4.1	1.58	1.02	121.2 ± 8.6	4.15	1.34
Parental	H292	NE	NE	18.8 ± 4.4	ND	ND	32.1 ± 2.1	ND	ND	40.5 ± 0.9	ND	ND
Gefitinib resistance	H292-GefR	†	††	24.8 ± 3.1	1.32	1.00	57.8 ± 5.6	1.80	1.00	51.2 ± 3.0	1.26	1.00
Parental	HEK293	NE	NE	10.0 ± 1.0	ND	ND	5.9 ± 1.1	ND	ND	5.0 ± 3.4	ND	ND
Empty vector transfected	HEK293/Vector	NE	NE	7.0 ± 2.0	ND	ND	8.2 ± 1.0	ND	ND	4.1 ± 0.7	ND	ND
ABCB1-GFP transfected	HEK293/ABCB1	††	†	21.6 ± 3.2	3.04	1.00	14.7 ± 1.7	1.78	1.00	20.0 ± 1.0	4.88	1.00
Developed cross-resistance (EV transfection + 5-FU)	HEK293/Vector/5-FU	NE	NE	10.1 ± 2.3	1.43	0.46	11.5 ± 0.5	1.40	0.78	5.5 ± 1.0	1.33	0.27
Developed cross-resistance (ABCB1-GFP transfection + 5-FU)	HEK293/ABCB1/5-FU	†††	†	54.2 ± 4.6	7.70	2.51	25.5 ± 1.2	3.10	1.74	44.8 ± 1.0	10.90	2.24

a Fold drug resistance versus parental cell line.

b Fold drug resistance versus PacR, GefR, or ABCB1-GFP transfected cells phenotype.

Abbreviations used: WB; Western blot, RR; relative resistance, †; low level expression, ††; medium level expression, †††; high level expression, NE; no expression, ND; not determined.

### Multiple cross-resistance in PTX-resistant cancer cells involves regulated *TUBB3* and *FOXO3a* expressions and supports escape from drug-induced apoptosis

**Figure 3:**
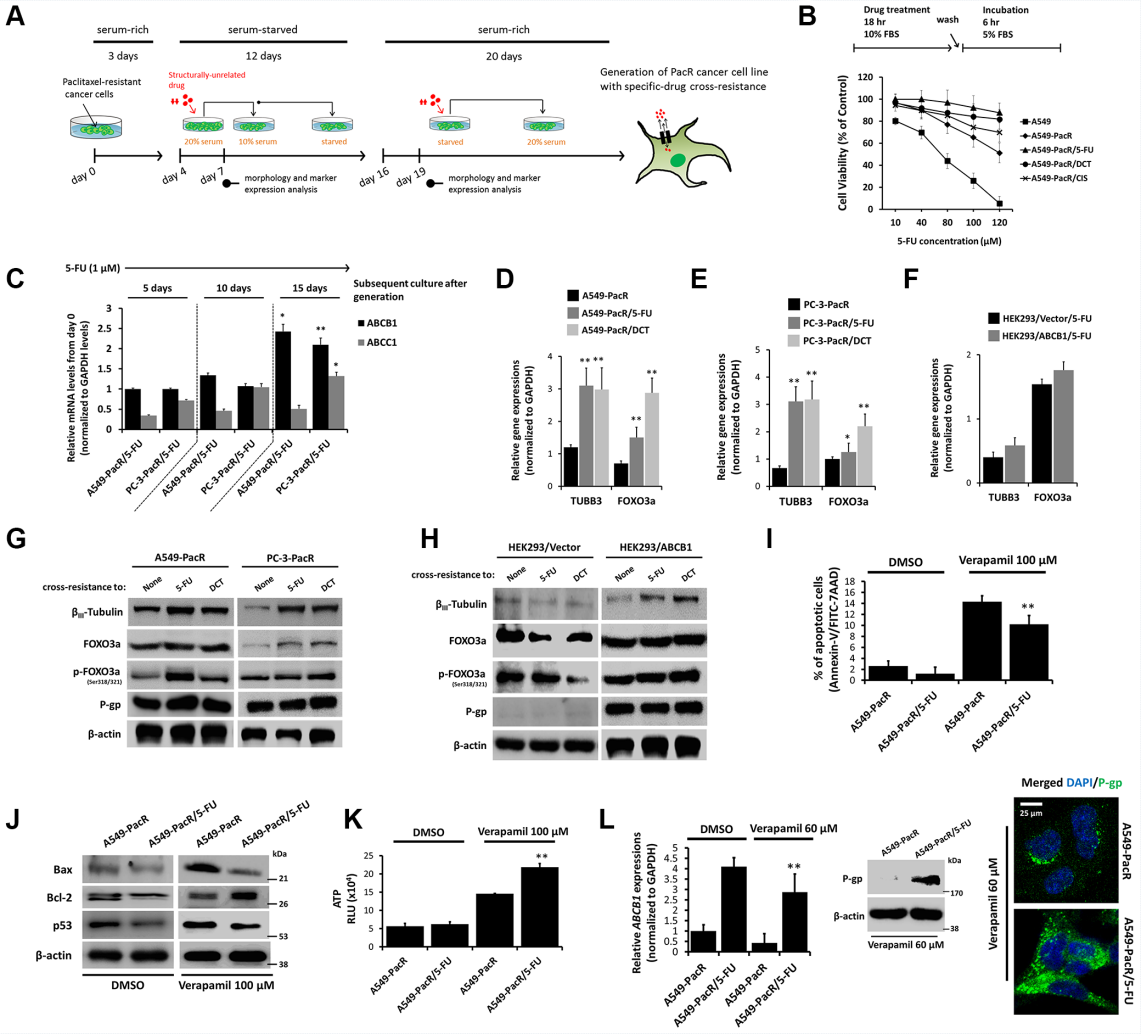
High acquired cross-resistance correlates to regulated TUBB3 and FOXO3a expressions with distinct hyperactive ABCB1 transcription in paclitaxel-resistant cancer cells (**A**) Schematic diagram of general strategy used for the generation of transient cross-resistance to 5-FU, DCT, or CIS in PacR phenotype cancer cells or *ABCB1*-GFP transfected HEK293 cells. (**B**) Growth rate response of indicated cells (lower panel) to 5-FU treatment in a dose-dependent manner. Schematic schedule of treatment is also displayed (upper panel). Cell viability was determined using MTT assay. Data are represented as means ± SEM. (**C**) Characterization for maintained *ABCB1* and *ABCC1* mRNA expressions in developed A549-PacR/5-FU and PC-3-PacR/5-FU cells after indicated subsequent cell cultures. Passages of cells were maintained with 1 μM 5-FU final concentration. (**D**–**F**) Characterization for *TUBB3* and *FOXO3a* mRNA expressions in indicated developed transient cross-resistance in PacR phenotype derived from A549 (D), PC-3 (E), and in developed transient 5-FU cross-resistance derived from HEK293 (F) cells. (**G** and **H**) Western blot analysis of A549, PC-3-PacR cells (G) and HEK293 cells transfected with either empty vector or *ABCB1*-GFP (H) cells, all with developed transient cross-resistance to indicated drugs. Cells were assessed for expressions of indicated proteins after 24 hr cell culture. (**I**) Flow cytometric determination of verapamil-induced apoptosis in indicated cells. Cells were treated with or without 100 μM verapamil for 24 hr. Data are shown as bar graph represented as means ± SEM. (**J**) Western blot analysis of indicated cells for expressions of apoptotic markers Bax, Bcl-2, and p53. Cells were treated with or without 100 μM verapamil for 24 hr. (**K**) Intracellular ATP level assessment in indicated cells. Cells were treated with or without 100 μM verapamil for 24 hr and ATP levels were determined in 10^4^ fraction of cells. RLU, relative luciferase units. Data are represented as means ± SEM. **(L**) Determination of verapamil-induced inhibition of P-gp/*ABCB1* in indicated cells assessed through qRT-PCR (left), Western blotting (center) and confocal microscopy (right). Cells were treated with 60 μM verapamil for 24 hr. Confocal images shown were magnified to 80 μm.

The overexpression of *TUBB3* has been clinically implicated in several malignancies with PTX-resistance [25]; this mechanism appears to be dependent on tubulin-mutation-induced decreases in mt stability [26]. Meanwhile, an *Akt*-dependent increase of *FOXO3a* activity was observed in response to PTX-induced apoptosis and was correlated with limited mt stability conferring *ABCB1*-associated drug resistance [15, 27, 28]. To study the potential phenomenon of *TUBB3* and *FOXO3a* regulation in the context of PTX-resistance and MDR in cancer cells, PTX-resistant cells with developed transient cross-resistance were analyzed (Figure 3A). Late-passage (p#21) cells were subjected to 5-FU treatment, which showed the growth inhibition in A549 parental cells in a concentration-dependent manner whereas A549-PacR cells with developed transient cross-resistance to 5-FU, DCT, and CIS displayed continued resistance even at a high concentration treatment with a higher fraction of cells surviving than just A549-PacR cells (Figure 3B). To associate this sustained cross-resistance with P-gp, *ABCB1* mRNA levels were determined in late-passage A549-PacR/5-FU and PC-3-PacR/5-FU cells while being maintained with very low concentrations of 5-FU overtime. The cells were harvested at days 0, 5, 10, and 15 subsequent to cell passage (reaching p#12–14) and subculture processes after generation and were analyzed for target gene expression. We found significantly increased levels of *ABCB1* over time (relative to day 0) that were sustained even at day 15 in both cells. Sustained transient cross-resistance to 5-FU in PTX-resistant cells appeared to involve *ABCC1* because the gene levels were slightly increased over time (Figure 3C). *TUBB3* and *FOXO3a* gene levels were significantly increased in A549-PacR/5-FU, A549-PacR-/DCT (Figure 3D), PC-3-PacR/5-FU, and PC-3-PacR-/DCT (Figure 3E) cells compared to the PacR phenotype only and were slightly regulated, although an increase was detectable, in HEK293/*ABCB1* cells compared to EV-transfected cells (Figure 3F). To confirm that the qRT-PCR data were correlated with increased protein levels, the expressions of β_III_-tubulin, phosphorylated FOXO3a, and P-gp were determined. Consistent with the findings of gene expression, the expressions of β_III_-tubulin and phosphorylated FOXO3a were up-regulated in 5-FU and DCT cross-resistant cells compared to the PacR phenotype only. In addition, all of the cells also exhibited continued P-gp expression (Figure 3G). In HEK293 cells, the establishment of transient cross-resistance to 5-FU and DCT in the *ABCB1*-overexpressing subline caused an activation of β_III_-tubulin compared to the minimally down-regulated levels in EV-transfected cells (Figure 3H). These data suggest that *TUBB3* and *FOXO3a* play significant roles in conferring cross-resistance in PTX-resistant cancers associated with *ABCB1*.

We observed that 5-FU (40 μM in form of pre-treatment) stimulated an escape from etoposide-induced apoptosis in A549-PacR cells (Supplementary Figure 1). To functionally examine whether P-gp-associated cross-resistance to 5-FU confers apoptosis protection, we employed the P-gp inhibitor, verapamil. A549-PacR/5-FU cells showed a reduced fraction of apoptotic cells compared to A549-PacR cells after treatment with an apoptosis-inducing concentration of verapamil (Figure 3I). Of note, this effect was supported by the expression of apoptotic biomarkers. Verapamil down-regulated the expressions of both Bax and p53 and up-regulated the expression of Bcl-2 in A549-PacR/5-FU cells compared to A549-PacR cells (Figure 3J). A rapid increase in verapamil-induced intracellular ATP levels was also observed in A549-PacR/5-FU cells (Figure 3K). Interestingly, 5-FU cross-resistance in PacR cells caused minimal deficiency in the verapamil-induced regulation of P-gp/ABCB1 with prevented rapid *ABCB1* gene suppression, but there was an observed reduction in A549-PacR/5-FU cells (left panel, Figure 3L). Compared to the A549-PacR cell line, from which the PacR/5-FU variant was derived, verapamil caused a failure to inhibit P-gp expression and distribution (right panel, Figure 3L). These results argue that the physiological role of MDR in protecting cells from apoptosis may have different genetic drivers addressing P-gp protection instead of the direct effects of drugs on P-gp. In support of this, it has been reported that PTX-resistance in breast cancer cells is associated with profound changes in the cell death response with the regulation of multiple apoptotic factors conferring platinum resistance [29].

### *FOXO3a* regulates *ABCB1* transcription to coordinate the *TUBB3* response in PTX-resistant cancer cells with 5-FU cross-resistance

**Figure 4:**
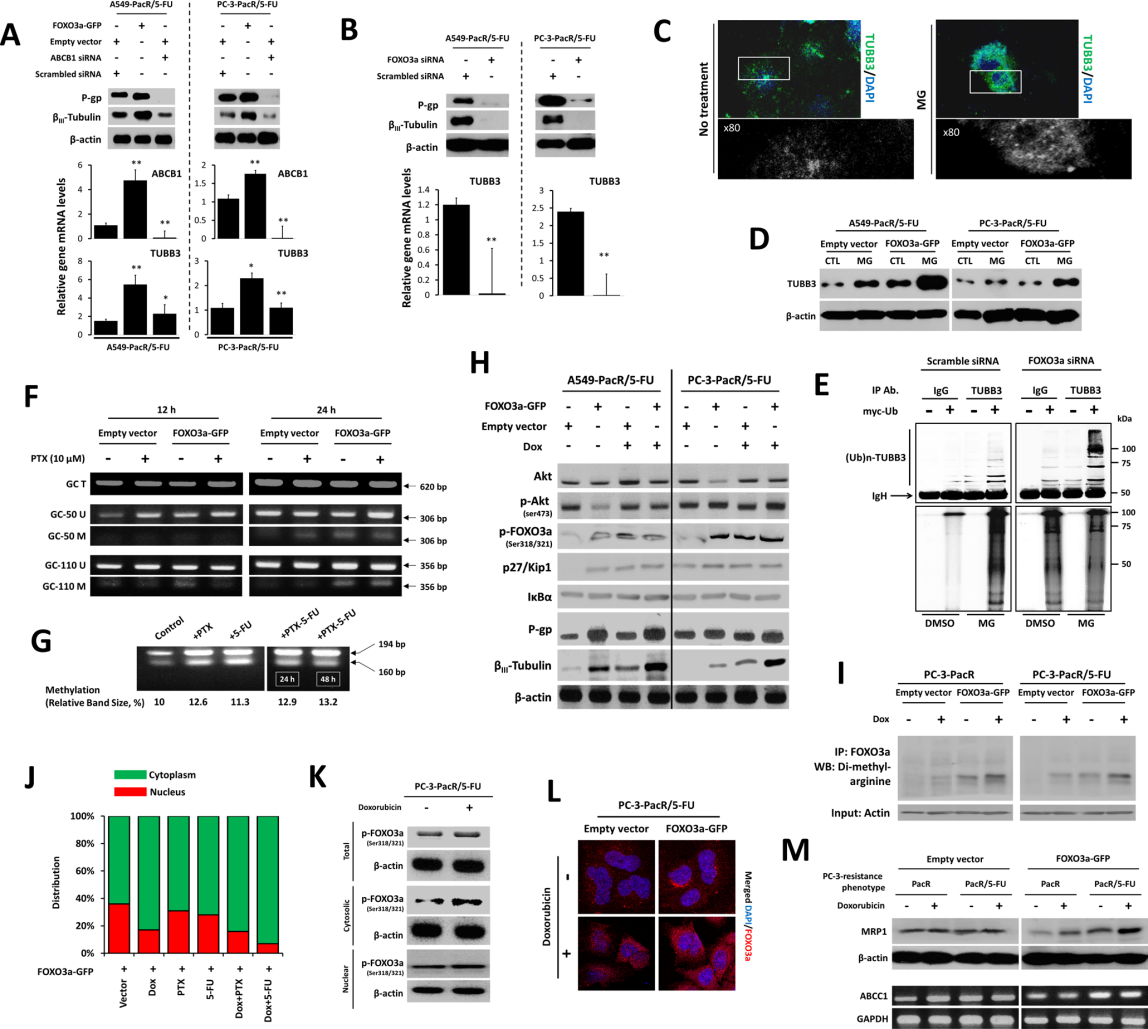
FOXO3a activity involves ABCB1 regulation to control TUBB3 response in paclitaxel-resistant cancer cells with transient 5-FU cross-resistance (**A**) Characterization of ABCB1 and TUBB3 expressions at both protein (upper panel) and mRNA (lower panel) levels after transient transfections with indicated vectors or siRNA in A549-PacR and PC-3-PacR both with developed 5-FU transient cross-resistance. Cells were transfected with empty vector or FOXO3a-GFP and scrambled siRNA or ABCB1 siRNA for 48 hr. Data are represented as means ± SEM. (**B**) Protein expressions (upper panel) of ABCB1 and TUBB3 and mRNA levels of TUBB3 (lower panel) in cells same as in A after transient transfection with either scrambled siRNA or FOXO3a siRNA for 48 hr. Data are represented as means ± SEM. (**C**) Effect of proteasome inhibitor, MG132 (MG), on β_III_-tubulin distribution. Cells treated with or without 50 μM MG for 24 h were subjected to immunocytochemistry. Magnified images were zoomed at ×80. The cells were stained with TUBB3 antibody and DAPI. (**D**) Effect of MG on TUBB3 protein expressions in same cells as in A. Cells were transfected with either empty vector or FOXO3a-GFP for 48 hr and treated with or without 50 μM MG132 for 24 hr. Whole-cell lysates were assayed by Western blotting. (**E**) Detection of ubiquitinated TUBB3 protein in A549-PacR/5-FU cells. Cells were transfected with either scramble siRNA or FOXO3a-siRNA for 48 hr and cells were further transfected with an empty vector or myc-tagged ubiquitin-encoding vector for 24 hr. Immunoprecipitation was performed by TUBB3 antibody, then ubiquitinated proteins were detected by myc antibody assayed by Western blotting. (**F**) Methylation-specific PCR of *ABCB1* -50GC and -110GC boxes in A549-PacR/5-FU cells. Primer sets are designed to amplify methylated (M) and unmethylated (U) alleles. A primer set encoding the whole GC region (T) was used as loading control. Cells were transiently transfected with either empty vector or FOXO3a-GFP for 48 hr and cells were treated with 10 μM PTX for 12 hr (left lane) or 24 hr (right lane). (**G**) Combined bisulphite restriction analysis of the Inr ABCB1 promoter region in A549-PacR/5-FU cells treated with or without 10 μM PTX, or 40 μM 5-FU for 24 hr or in combination for 24 or 48 hr as indicated. Figures represent the methylation percentages observed in the indicated drug-treated cells obtained from two separate independent experiments. (**H**) Effect of doxorubicin (Dox) on FOXO3a-induced regulation of Akt-related signals. Cells were transiently transfected with either empty vector or FOXO3a-GFP for 48 hr and cells were treated with or without 2 μM Dox for 8 hr. Protein expression levels were analyzed by Western blotting. (**I**) Methylated FOXO3a status in PC-3-PacR and –PacR/5-FU cells. Cells were transiently transfected with either empty vector or FOXO3a-GFP for 48 hr and cells were treated with or without 2 μM Dox for 8 hr. Cell lysates were taken and dimethylated proteins at arginine residues were immunoprecipitated using anti-human FOXO3a antibody followed by Western blotting using anti-dimethyl arginine antibody. Actin was used as input. (**J**) Average GFP-positive cells determination in the nucleus or cytoplasm of PC-3-PacR/5-FU cells. Cells were transiently transfected with FOXO3a-GFP for 48 hr followed by treatment with or without 2 μM Dox, 300 nM PTX, 120 μM 5-FU or their indicated combinations for 8 hr. (**K**) Lysates of PC-3-PacR/5-FU treated with or without 2 μM Dox for 8 hr were subjected to cytoplasmic or nuclear extraction followed by Western blotting. (**L**) Confocal microscopic analysis of PC-3-PacR/5-FU cells. Cells were transiently transfected with either empty vector or FOXO3a-GFP and cells were treated with or without 2 μM Dox for 8 hr and stained with FOXO3a antibody and DAPI. Images shown were magnified at 60 μm. (**M**) Characterization of ABCC1 expressions at both protein (upper panel) and gene transcript (lower panel) levels in both PC-3-PacR and –PacR/5-FU cells. Cells were transiently transfected with either empty vector or FOXO3a-GFP for 48 hr and cells were treated with or without 2 μM Dox for 8 hr.

To determine the mechanism by which regulated *FOXO3a* and *TUBB3* levels affect P-gp-associated MDR in PTX-resistant cancer cells, we employed transient GFP-tagged-gene and/or siRNA transfections (see the Experimental Procedures) in A549-PacR/5-FU and PC-3-PacR/5-FU cells. The difficulty of identifying *TUBB3* effector genes and other interacting factors has been addressed in drug-resistant cancers because of the complexity of tubulin auto-regulation many years before identifying the association of β-tubulin isotypes and PTX resistance [30]. *FOXO* transcription factors regulate mt defects by regulating tubulin loops [19, 31]; although this observation has not yet been implicated in several malignancies, considerable progress has been made in associating FOXO-mediated mt stability regulation and taxol-resistance in some cancers. In A549-PacR and PC-3-PacR sublines with 5-FU cross-resistance, the overexpression of *FOXO3a* up-regulated both *ABCB1* and *TUBB3* whereas the direct silencing of *ABCB1* caused incomplete inhibition of *TUBB3* at both mRNA and protein levels (Figure 4A). To determine whether *FOXO3a* is able to directly regulate *TUBB3*, the *FOXO3a* gene was knocked down, and silencing *FOXO3a* was shown to directly inhibit *TUBB3* with both mRNA and protein expression completely suppressed (Figure 4B).

The ubiquitin-proteasome system has also been associated with β_III_-tubulin protein degradation and epigenetic regulation [32]. To further dissect how β_III_-tubulin protein expression is controlled in cancer cells with MDR, we employed the ubiquitin-proteasome-dependent degradation pathway. When A549-PacR/5-FU and PC-3-PacR/5-FU cells transfected with EV or *FOXO3a*-GFP are treated with the proteasome inhibitor, MG132, it suppresses the degradation of β_III_-tubulin in both cell models; furthermore, *FOXO3a* enhances β_III_-tubulin activation with an observed 3-fold increased accumulation of *TUBB3* turnover (Figure 4C, 4D). This finding suggests that the effect induced by MG132 pin-points the constitutive degradation of TUBB3 through its ubiquitination status. We further examined the status of TUBB3 poly-ubiquitination in these cells by transfecting the myc-tagged ubiquitin expression vector. After anti-TUBB3 immunoprecipitation followed by anti-myc antibody detection, we detected strikingly high molecular weight smears following MG132 treatment with higher polyubiquitinated levels in *FOXO3a*-siRNA transfected-cells relative to EV transfected cells, while lower poly-ubiquitination status in non-treated controls (Figure 4E). These results demonstrate that FOXO3a strikingly influences the physiological control of β_III_-tubulin, at least in part, by the ubiquitin-proteome system, which can potentially modulate cross-resistance to 5-FU in PTX-resistant cancer cells.

Frequent aberrant DNA methylation of *ABCB1* has been observed in highly drug-resistant and invasive cancers, making its hypomethylation-triggering factors desirable targets for transcription-based inhibition, including the regulation of histone acetylation to reverse MDR [33, 34]. However, pivotal effectors that regulate *ABCB1* methylation and their precise epigenetic mechanisms have not yet been well elucidated. To determine whether the DNA methylation status of *ABCB1* is influenced by *FOXO3a* to effect PTX-resistance and MDR, we amplified our designed primers specific to both methylated and unmethylated alleles of the -50GC and -110GC boxes of *ABCB1*. Among the several GC boxes of *ABCB1* that are essential for its activation, the -50GC and -110GC boxes are regarded to be highly relevant binding sites for *ABCB1* activators and repressors [35]. The -50GC box appeared to be slightly hypomethylated in 12 hr culture whereas it displayed detectable methylation in 24 hr culture, which was emphasized when the cells overexpressed *FOXO3a* and received PTX treatment. The -110GC box was determined to have relatively higher methylation levels with the same observed status as that of the -50GC box when the cells overexpressed FOXO3a in 24 hr culture (Figure 4F). To further confirm that the methylation modifications in PTX-resistant cells with 5-FU cross-resistance were stimulated in part by drugs, we employed a COBRA analysis of the cytosine-phosphate-guanine (CpG) site of the Inr region of *ABCB1*. DNA methylation was increased after a relatively higher dose of PTX, 5-FU, and combination treatments (Figure 4G). Thus, the changes in *ABCB1* promoter methylation can be triggered partly by *FOXO3a* overexpression and drugs, whereby cancer cells have acquired first-line resistance and cross-resistance.

To examine whether the *FOXO3a*-mediated regulation of *ABCB1* and subsequent *TUBB3* expression are influenced by *FOXO3a* up-stream effectors, we determined the expressions of Akt signaling molecules that were highly associated with FOXO3a activity. We also employed doxorubicin to mechanistically identify the role of drug-induced FOXO3a phosphorylation on P-gp expression because doxorubicin was found to induce Akt-dependent FOXO3a activity [36]. Doxorubin up-regulated Akt and FOXO3a and increased their phosphorylation in both A549-PacR/5-FU and PC-3-PacR/5-FU cells. FOXO3a overexpression derived more preserved doxorubicin-induced p27/Kip1 activation except for IκB-α, which appeared to be unaffected in both conditions. Doxorubicin strikingly up-regulated P-gp and β_III_-tubulin expression in cells overexpressing FOXO3a (Figure 4H). Given that PI3K/Akt signaling is involved in predominant MDR, which is further associated with the increase of doxorubicin-mediated FOXO3a nuclear accumulation [37], these findings suggest that the involvement of *FOXO3a* activity in regulating the P-gp and β_III_-tubulin response involves a doxorubicin-induced PI3K/Akt mechanism. Interestingly, we found that in both drug-resistant phenotypes of the PC-3 cell line, PacR and PacR/5-FU, doxorubicin stimulated the methylation of FOXO3a at the arginine residues of the cells overexpressing FOXO3a (Figure 4I). This suggests that an alteration in the Akt-induced phosphorylation inhibition of FOXO3a through arginine methylation is associated with acquired resistance. We then studied whether the doxorubicin-induced nuclear translocation of FOXO3a is affected using PC-3-PacR/5-FU as a model. Notably, we found an increase in cytosolic FOXO3a when cells received doxorubicin alone or in combination with either PTX or 5-FU (Figure 4J, 4K), with doxorubicin alone causing cytosolic condensation of FOXO3a in the same cells (Figure 4L).

We next addressed whether MRP1 (encoded by *ABCC1*) expression is involved in the *FOXO3a*-regulated *ABCB1* mechanism in the PC-3-PacR/5-FU phenotype because this specific model was found to overexpress MRP1 at higher levels compared to the same line of acquired resistance derived from A549 cells (see Table 1). We found no significant changes, although MRP1 is abundantly expressed in EV-transfected cells, whereas in FOXO3a overexpressing cells, doxorubicin induced MRP1 up- regulation in both protein and gene expression levels (Figure 4M). Similar to previous reports, this finding may support the high correlation of MRP1 hyperactivation with PTEN-controlled PI3K/Akt cascade in acquired drug resistance in prostate cancer cells [38].

### Drug-induced secretome factors promote FOXO3a-regulated P-gp function to confer multiple cross-resistance in PTX-resistant cancer cells

**Figure 5:**
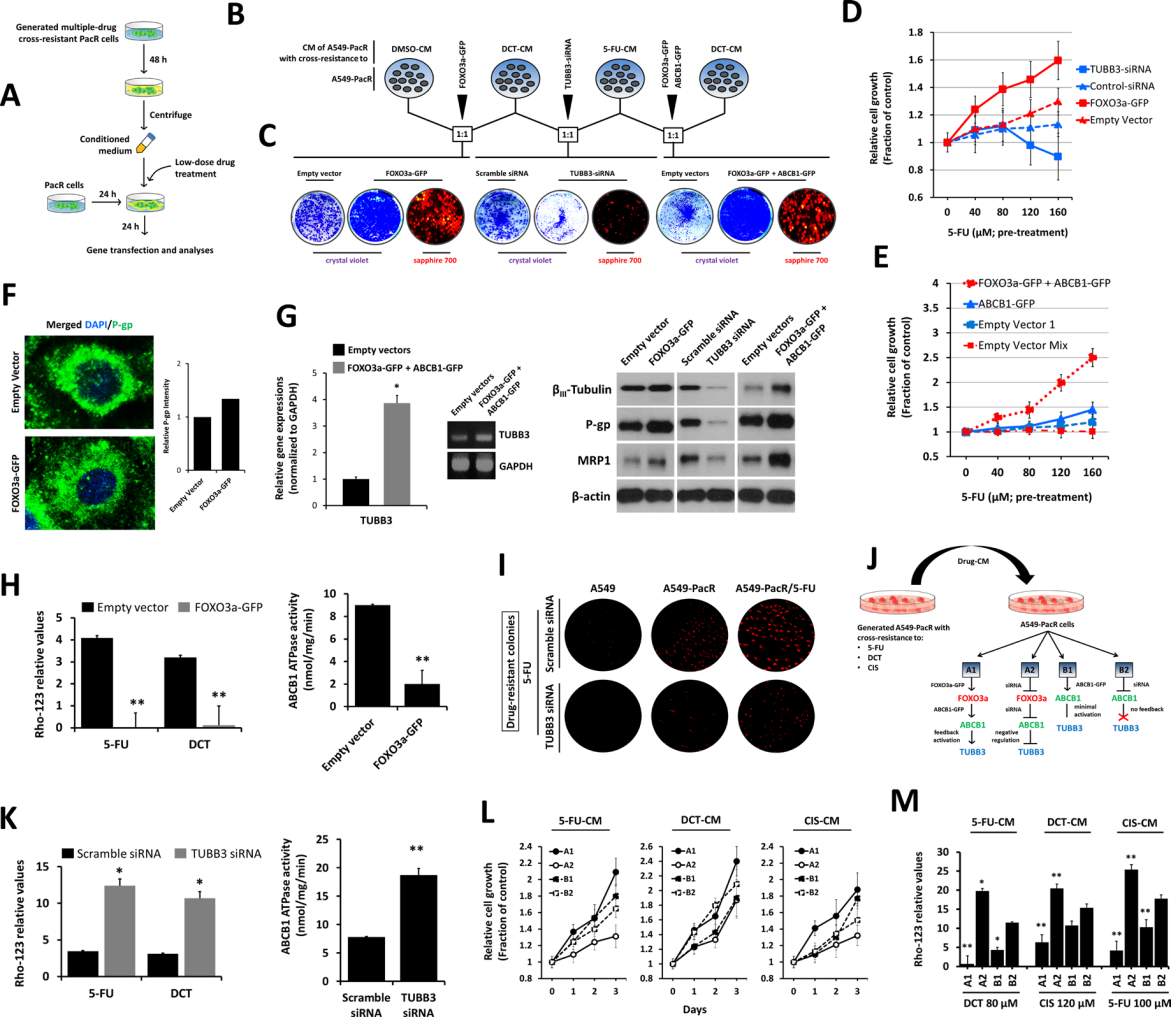
Drug-induced secretome factors influence MDR promotion of FOXO3a-regulated P-gp activity in PTX-resistant A549 cells with multiple cross-resistance (**A**) Schematic diagram of the drug conditioned media (CM)-exposure procedure used in various assays (**B**–**J**). (B and C) Characterization of FOXO3a-mediated multiple cross-drug resistance in A549-PacR cells (B). Cells were preincubated with indicated 1:1 combination ratio of CM from DMSO-, DCT-, 5-FU-treated cells. After 24 hr, cells were transiently transfected with indicated vectors or siRNA for 48 hr and assessed for colony formation (C). Established drug resistant colonies were stained and visualized by crystal violet or sapphire 700. (D and E) TUBB3 knockdown or FOXO3a overexpression (D) and overexpression of ABCB1 and FOXO3a effects on growth rate of A549-PacR/5-FU cells (E). A549-PacR/DCT cells were treated with indicated 5-FU concentrations for 24 h then CM was collected. Adherent A549 cells were exposed to the collected CM for 24 h followed by transient transfection with indicated vectors or siRNA for 48 hr. Cell viability was assessed by MTT assay. Data are represented as means ± SEM. (F) Confocal microscopic analysis of A549-PacR/5-FU cells. Cells were transiently transfected with indicated vectors or siRNA for 48 hr and stained with P-gp antibody and DAPI. Images shown were magnified to 80 μm. (G) Western blot analysis (left) and agarose gel electrophoresis of qPCR products of A549-PacR/5-FU cells. Cells were transiently transfected with indicated vectors or siRNA for 48 hr and assessed for expression studies. (H) ABCB1 drug efflux activity of A549-PacR/5-FU cells. Cells were transiently transfected with either empty vector or FOXO3a-GFP for 48 hr and cells were treated with or without 100 μM 5-FU or 120 μM DCT for 24 hr. Drug-treated cells were assayed for Rhodamine-123 (Rho-123) uptake (left) and untreated cells were assayed for ABCB1 ATPase activity (right). Data are represented as means ± SEM. (I) ABCB1 drug efflux activity of A549-PacR/5-FU cells. Cells were transiently transfected with either scrambled siRNA or TUBB3 siRNA for 48 hr followed by drug treatment and assayed as in H. Data are represented as means ± SEM. (J) Multidrug resistant colonies. Cells were transiently transfected with either scramble siRNA or TUBB3 siRNA for 48 hr and cells were treated with 30 μM 5-FU for 7 days. Resistant colonies were visualized by sapphire 700. (**K**) Schematic diagram of drug CM exposure and respective TUBB3 feedback after induced gene expression changes via transient gene transfection or siRNA gene silencing. Indicated gene or siRNA transfection schemes were used in L and M for P-gp functional assays. (**L**) Growth rate of A549-PacR/5-FU cells. Cells were pre-incubated with indicated drug-CM and cells were transiently transfected with indicated genes or siRNA for 48 hr. Cell viability was assessed by MTT assay. Data are represented as means ± SEM. (**M**) ABCB1 drug efflux activity A549-PacR/5-FU cells. Cells were pre-incubated with indicated drug-CM and cells were transiently transfected with indicated genes or siRNA for 48 hr. Cells were then treated with indicated drugs and concentration for 36 hr. Data are represented as means ± SEM.

Cancer growth and therapy resistance have been shown to be strongly regulated by soluble mediators from complex microenvironments that consist of secreted signals from immune, stromal, and cancer cells [39, 40]. Recent findings have suggested that signals derived from sensitive cancer cells in response to targeted therapies, such as kinase inhibitors, drive the outgrowth of drug-resistant cells [41]. Based on these findings and to refocus on cross-resistance manifestation, we hypothesized that signals derived from PTX-resistant cancer cells with cross-resistance to 5-FU and the PTX-analogue, DCT, can influence the contentious onset of multiple cross-resistance in first-line PTX-resistant cancers. To examine this speculation *in vitro*, we acquired conditioned media (CM) (see schematic diagram in Figure 5A) from PacR/5FU, PacR/DCT, and their combination (1:1 ratio) cultured with low-dose drug treatment and co-cultured this media with young passage A549-PacR cells (see schematic diagram in Figure 5B). We mixed CM from A549-PacR/DCT with DMSO-treated cell CM and performed subsequent *FOXO3a* gene transfections; we combined A549-PacR/DCT-CM with A549-PacR/5-FU-CM and performed subsequent *TUBB3* RNAi; and we combined A549-PacR/5-FU-CM with A549-PacR/DCT-CM and performed dual *FOXO3a* and *ABCB1* gene transfections. The rationale behind these combinations was based on previous findings relating the gene of interest to a specific class of cross-resistance occurring in drug-resistant cancers [42, 43, 44]. To study the effect of the above-described scheme on the *FOXO3a*-regulated *ABCB1* mechanism and *TUBB3* response in a more growth-relevant system, we performed a colony formation assay. PacR cells pre-exposed to DCT-CM following *FOXO3a*-GFP transfection formed more drug-resistant colonies than EV-transfected cells. The transient knockdown of *TUBB3* prevented the outgrowth of colonies derived from PacR cells pre-exposed to a combination of 5-FU-CM and DCT-CM, producing significantly lesser colonies than scramble control cells. Furthermore, the effect of *TUBB3* knockdown on colony formation with the same drug combination-CM pre-exposure was completely opposite that of cells overexpressing both *FOXO3a* and *ABCB1* (Figure 5C), whereas the observation was significantly supported with the cell growth response of A549-PacR cells that experienced the same drug-CM pre-exposure as described above (A549-PacR/5-FU-CM) with subsequent dose-dependent 5-FU treatment. Relative to cells transfected with either EV or scramble siRNA, *FOXO3a* overexpression promoted drug-resistant cell growth whereas the knockdown of *TUBB3* suppressed cell growth in a dose-dependent manner (Figure 5D). To determine whether *FOXO3a*-induced growth promotion involves *ABCB1*, we transfected the same cells with *ABCB1*-GFP alone, co-transfection with *FOXO3a*-GFP, or transfection with their respective empty vectors. The cells overexpressing both *FOXO3a* and *ABCB1* proliferated more rapidly than the cells overexpressing *ABCB1* alone following 5-FU treatment (Figure 5E). Consistent with the involvement of *ABCB1*, A549-PacR cells that were pre-exposed to A549-PacR/5-FU-CM followed by subsequent *FOXO3a*-GFP transfection displayed higher P-gp distribution than control cells (Figure 5F). Employing the same scheme of drug-CM exposure, transient gene and/or siRNA transfections as in Figure 5B, we found regulated gene expressions of *TUBB3* (left panel) and protein expressions of β_III_-tubulin, P-gp, and MRP1 (right panel) in A549-PacR cells, suggesting that the secreted factors from 5-FU cross-resistant PacR cells influence the promotion of MDR through a *FOXO3a*-directed *ABCB1* mechanism (Figure 5G). Notably, in the same cells, the overexpression of either *FOXO3a* or both *FOXO3a* and *ABCB1* subsequently induced the activation of MRP1.

To functionally characterize the effects of *FOXO3a* on P-gp activity, we addressed the drug efflux function of the P-gp pump incorporating the Rho-123 and ABCB1 ATPase assays. *FOXO3a* overexpression significantly blocked the intracellular accumulation of the Rho-123 fluorescent substrate following 5-FU and DCT treatment, which was supported by the suppression of ABCB1 ATPase activity (Figure 5H left panel). Correspondingly, *TUBB3* knockdown resulted in enhanced accumulation of the substrate and increased ABCB1 ATPase activity (Figure 5H right panel). Accordingly, *TUBB3* knockdown sensitized the cells to 5-FU, resulting in fewer formed colonies compared to control cells upon drug exposure (Figure 5I).

To understand the role of secreted signals from A549-PacR cells with multiple acquired cross-resistance in promoting P-gp-associated MDR, we directly harvested CM from A549-PacR/5-FU, A549-PacR-/DCT, and A549-PacR-/CIS cells followed by individual or combination transient gene or siRNA transfections (see scheme in Figure 5J). The *TUBB3* gene response to various transfections was determined by qRT-PCR (Supplementary Figure 2) and was used as an indicative mechanism factor in consequent cell growth and Rho-123 accumulation. Drug-resistant cells progressed significantly more rapidly when *TUBB3* was activated in response to co-transfection of *FOXO3a*-GFP and *ABCB1*-GFP whereas cell growth progressed more slowly when *TUBB3* was negatively regulated in response to co-transfection of *FOXO3a*-siRNA and ABCB1-siRNA (Figure 5L). Verifying this finding, Rho-123 intracellular accumulation was inhibited when *TUBB3* was suppressed whereas Rho-123 substrate accumulation was increased when *TUBB3* was activated in response to similar gene modifications as described above (Figure 5M). The results of the cell growth and Rho-123 accumulation assays for cells transfected with gene/siRNA respective controls (EV, scramble siRNA) can be observed in Supplementary Figure 3; although measurable, no significant differences were found. Taken together, these data indicate that secretome factors from PTX-resistant cancer cells with acquired cross-resistance can sufficiently influence MDR in first-line PTX-resistant cancers cells with impaired FOXO3a-regulated ABCB1 activity and can be predicted by *TUBB3* feedback.

Meanwhile, the above described regulatory effects of transient *TUBB3* knockdown on P-gp function may not be translated to GEF resistance because the reversal of drug resistance after siRNA-mediated silencing was not observed in H292-GefR cells (Supplementary Figure 4A, 4B). Notably, *TUBB3* knockdown did not significantly affect P-gp efflux and ATPase activities (Supplementary Figure 4C, 4D), which can be traced on the preserved *ABCB1* and *ABCC1* mRNA levels even after 5-FU and DCT stimulation (Supplementary Figure 4E, 4F, 4G). These results suggest that modification of *TUBB3*-directed effects on P-gp regulation and cross-resistance can only be implied to PTX-resistant cancer cells.

### Transient knockdown of *TUBB3* supresses the *ABCB1*-associated aggressive cell phenotype and alters subcutaneous tumor physiology

**Figure 6:**
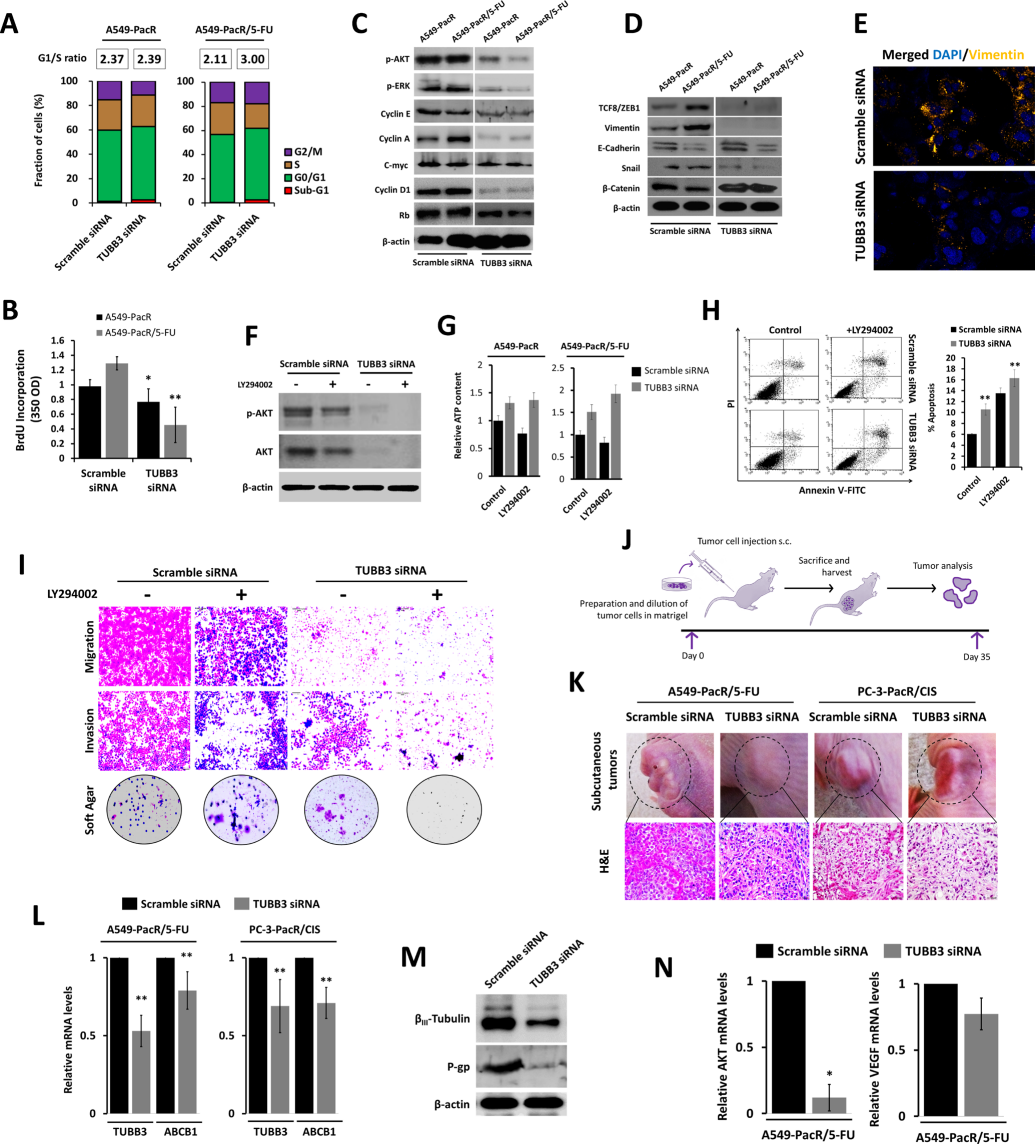
TUBB3 deletion involves AKT to alter the aggressive phenotype and tumor physiology of paclitaxel-resistant cancer with developed transient multiple cross-resistance (**A** and **B**) Cell cycle progression of A549-PacR and -PacR/5-FU cells. Cells were transiently transfected with either scramble siRNA or TUBB3 siRNA for 48 hr. Transfected cells were subjected to FACS analysis (A) and BrdU incorporation assay (B). G1/S ratio of cells was calculated and displayed. Data are represented as means ± SEM. (**C**) Cell cycle regulation in A549-PacR and -PacR/5-FU cells. Cells were transiently transfected with either scramble siRNA or TUBB3 siRNA for 48 hr. Cells were then subjected to Western blotting to detect cell cycle regulatory proteins. (**D**) Epithelial-to-mesenchymal transition (EMT) status of A549-PacR and -PacR/5-FU cells. Cells were transiently transfected with either scramble siRNA or TUBB3 siRNA for 48 hr. Classical EMT markers were immunoblotted. (**E**) Immunofluorescence images of Vimentin in A549-PacR/5-FU cells. Cells were transiently transfected with either scramble siRNA or TUBB3 siRNA for 48 hr. Cells were stained with Vimentin antibody and DAPI. Images shown were magnified at 30 μm. (**F**) Inhibition of AKT in A549-PacR/5-FU cells. Cells were transiently transfected with either scramble siRNA or TUBB3 siRNA for 48 hr and cells were treated with or without 10 μM of AKT inhibitor, LY294002, for 18 hr. Phosphorylation of AKT was assayed by Western blotting. (**G**) Intracellular ATP assessment of A549-PacR and -PacR/5-FU cells. Cells were transiently transfected with either scramble siRNA or TUBB3 siRNA for 48 hr and cells were treated with or without 10 μM LY294002 for 18 hr. ATP levels was assessed. Data are represented as means ± SEM. (**H**) AKT-dependent apoptosis in A549-PacR/5-FU cells. Cells were transiently transfected with either scramble siRNA or TUBB3 siRNA for 48 hr and cells were treated with or without 10 μM LY294002 for 18 hr. Cells were analyzed for annexin-V/FITC-PI by FACS. Data are represented as means ± SEM. (**I**) Motility and colony formation of A549-PacR/5-FU cells. Cells were transiently transfected with either scramble siRNA or TUBB3 siRNA for 48 hr and cells were treated with or without 10 μM LY294002 for 18 hr in culture subjected to migration, invasion, and colony formation assays. (**J**) Schematic diagram of *in vivo* tumor xenograft experiments. (**K**) Evaluation of subcutaneous tumors. Indicated cells were transiently transfected with either scramble siRNA or TUBB3 siRNA for 48 hr. Transfected cells (1 × 10^6^ cells/flank) were subcutaneously injected into the flanks of nude mice. Shown representative images depict the actual tumors prior to surgery and measurement (upper) and H&E (lower) immunohistochemical analysis indicated xenograft tumors. Stained sections were photographed with an inverted phase-contrast microscope with 150× magnification. (**L**) Relative expressions of ABCB1 and TUBB3 in tumor transcripts of indicated xenografts. Total RNA was isolated and analyzed by quantitative reverse transcriptase-PCR using ABCB1-, or TUBB3-specific primers and normalized to GAPDH expression. Data are represented as means ± SEM. (**M**) Protein expressions of β_III_-tubulin and P-gp in A549-PacR/5-FU xenograft tumors. Tumor sample lysates were immunoblotted with the indicated antibodies. (**N**) Relative expressions of AKT and VEGF in tumor transcripts of A549-PacR/5-FU. RNA was processed as in L. Data are represented as means ± SEM.

PTX-resistant cancers highlight the acquisition of an aggressive phenotype exhibiting abnormal nuclear morphology and faster cytoskeletal remodeling dynamics [43, 44]. To elaborate the involvement of *TUBB3* in the mechanism by which acquired PTX-resistance and 5-FU cross-resistance develop aggressiveness, we employed various cell-based and protein expression assays that characterize cancer cell invasiveness. In both A549-PacR and A549-PacR/5-FU developed phenotypes, *TUBB3* knockdown induced an increase in the G_0_/G_1_ transition phase, resulting in an increased G_1_/S-phase ratio compared to both controls (Figure 6A). Similarly, scant *TUBB3* transcription resulted in an expedited S-phase fraction identified by BrdU incorporation, down-regulated the phosphorylation of Akt and ERK, and promoted the inhibition of cell-cycle regulatory proteins, thus collectively explaining the gradual decrease in cell growth (Figure 6B, 6C). In relation, our preliminary findings on 5-FU stimulated outgrowth of A549-PacR cells suggest that *TUBB3* deficiency reduces the cells contentious growth rate but fails to induce complete attenuation (Supplementary Figure 5). Targeted silencing of *TUBB3* regulated the protein expressions of EMT markers, revealing a general trend away from the epithelium and towards the mesenchyme (Figure 6D). Interestingly, the distribution of vimentin in A549-PacR/5-FU control cells was condensed in the cytoplasm, and some appeared to be atypical to the nucleus. The same cells with silenced *TUBB3* expression inhibited the immunofluorescence of vimentin (Figure 6E). Along with Akt and ERK signals, the classical EMT markers encompass key factors that regulate drug-resistant aggressiveness [45]. This may suggest that modification of mt stability through *TUBB3* affects the distinctive EMT-driven hostile outgrowth of the tumor inter-cellular system in cancers with MDR, at least *in vitro*.

We next elucidated the probable involvement of Akt by incorporating the LY294002 inhibitor. The knockdown of *TUBB3* escalated the LY294002-induced Akt-inhibition in A549-PacR/5-FU cells at the protein level compared to scramble siRNA-transfected cells except with little alteration between non-treated and LY294002-treated siTUBB3-transfected cells (Figure 6F). In addition, a concurrent increase in intracellular ATP levels was evident in siTUBB3-transfected cells compared to scramble siRNA-transfected cells in both A549-PacR and A549-PacR/5-FU cells (Figure 6G). This prompted us to further evaluate the association of *TUBB3*-induced Akt activity suppression in cell mobility and invasiveness. The same attenuation of *TUBB3* siRNA-induced effects were observed in the Akt-dependent promotion of intrinsic apoptosis, which supports escape from drug-induced apoptosis (Figure 6H), and in cell migration, invasion, and colony formation in soft agar, indicating that the metastatic aggressiveness of PTX-resistant cells with acquired cross-resistance, at least to 5-FU, can be controlled by TUBB3 (Figure 6I).

In support of the identified *TUBB3*-dependent effects in multidrug resistance *in vitro*, a tumor xenograft model was used to examine the probable drug resistant-tumor physiological alteration *in vivo* by modifying *TUBB3* transcription. The nude mice were randomized and subcutaneously engrafted with A549-PacR/5-FU and PC-3-PacR/5-FU cells transfected *in vitro* with either scramble siRNA or *TUBB3* siRNA. At the termination of the experiment (35 days after inoculation) (see *in vivo* experiment scheme in Figure 6J), the TUBB3 deficient tumors showed less condensed tumor mass and induced susceptibility to apoptosis (Figure 6K). These data confirmed that the described genetic and other substantial factors affecting *TUBB3* transcription directly affect metastatic tumor progression of drug-resistant cancer cells, specifically PTX-resistant tumors with acquired cross-resistance to unrelated drugs (at least to 5-FU and CIS). Preliminarily, we observed the regression of tumor growth using a small-size sample group in *TUBB3*-deficient subcutaneous tumors. The subcutaneous tumor mass (in grams; g) of the control group was approximately 1.98 g (*n* = 2; 8 isolated tumor xenografts of A549-PacR/5-FU) and 0.89 g (*n* = 2; 8 isolated tumor xenografts of PC-3-PacR/CIS), whereas the mass of the tumor induced by TUBB3 siRNA-transfected cells was inhibited by 19% (*p* < 0.011) and 32% (*p* < 0.005) with tumor masses of approximately 1.62 g (*n* = 2; 8 isolated tumor xenografts of A549-PacR/5-FU) and 0.61 g (*n* = 3; 12 isolated tumor xenografts of PC-3-PacR/CIS) (Supplementary Figure 8), respectively.

A biochemical analysis of the tumor tissues was performed to substantiate the *in vitro* findings and evaluate those findings in a more physiologically-relevant manner. We confirmed the *in vivo* transient siRNA transfection efficiency by qRT-PCR using RNA extracted from tumor samples. *TUBB3* siRNA reduced the *TUBB3* mRNA level by 47% and the *ABCB1* mRNA level by 19% in A549-PacR/5-FU-induced subcutaneous tumors whereas in PC-3-PacR/CIS tumors, *TUBB3* was reduced by 31% and *ABCB1* by 29% relative to the mRNA levels from tumors induced by scramble siRNA transfected cells (Figure 6L). To further elaborate the consequences of scant *TUBB3* on P-gp expression, we determined their protein expression levels in extracts from frozen xenograft tumor tissue samples. We observed down regulated levels of β_III_-tubulin and P-gp in *TUBB3*-deficient subcutaneous tumors. We then determined the gene expressions of *Akt* and *VEGF* in an attempt to explain their effects on tumor progression. A similar paradigm in which tumor endothelial cells acquire drug resistance involving Akt and VEGF signaling to promote metastatic tumor growth was previously reported [46]. Thus, reduced tumor mass in response to *TUBB3* knockdown is influenced by Akt and VEGF signals, which we found to suppress *Akt* gene expression and attenuate *VEGF* levels in A549-PacR/5-FU tumors (Figure 6N). These results confirm the regulation of Akt signaling in modified *TUBB3* status associated with acquired cross-resistance in PTX-resistant cancer *in vivo* and *in vitro*.

### FOXO3a-directed feedback control of TUBB3 transcription confines PTX-targeted microtubule stability in PTX-resistant A549 cells with acquired cross-resistance

**Figure 7:**
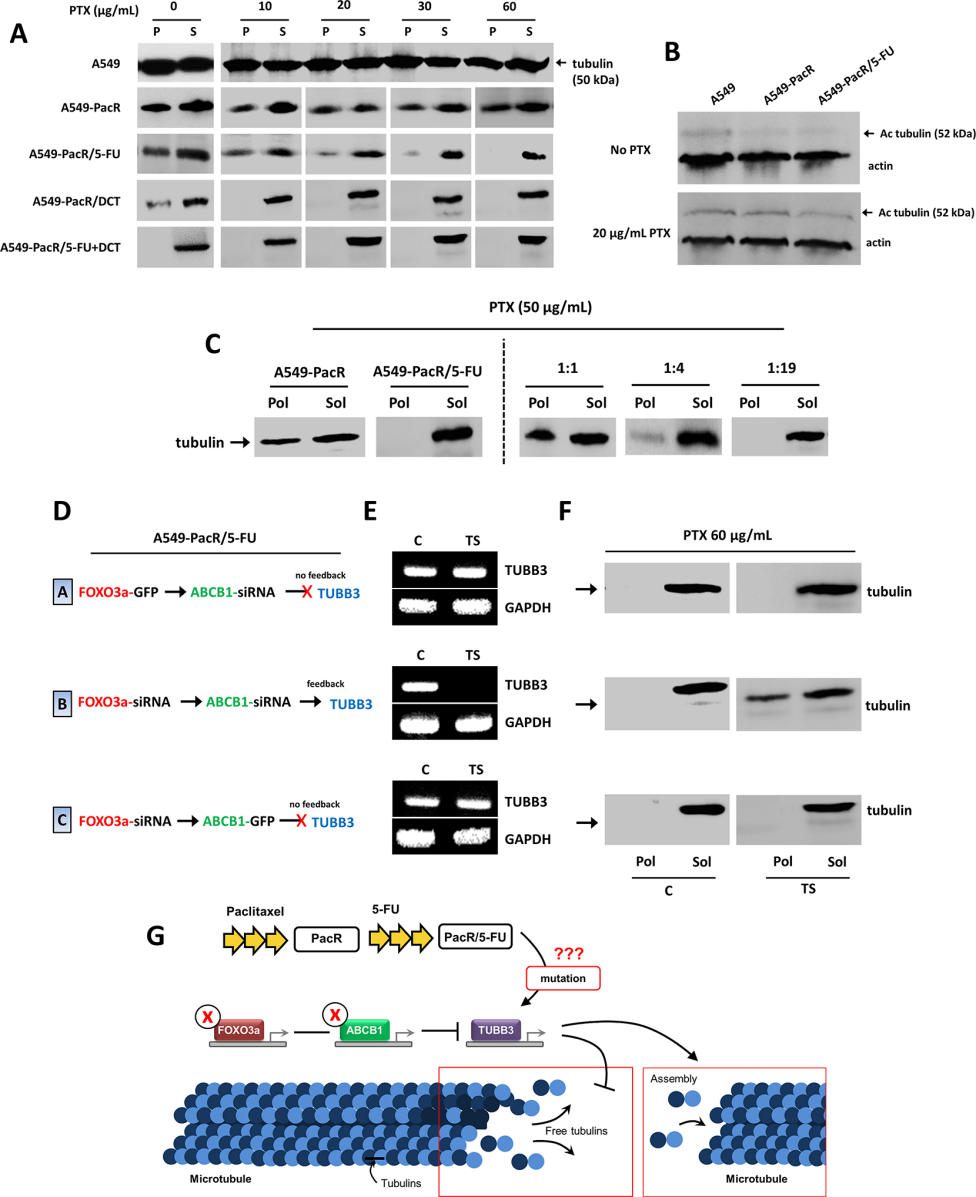
TUBB3 feedback inactivation reverses impaired microtubule stability in A549-PacR cells with developed transient multiple cross-resistance (**A**) Microtubule stability in parental, PacR, and developed PacR phenotype cells with multiple transient cross-resistance. Cells were grown in the presence of indicated PTX concentrations for 18 hr. Following cell lysis, pellet (P) and the supernatant (S) protein fractions were separated by centrifugation and resolved on adjacent lanes by electrophoresis. Transferred filters were probed with tubulin antibody. (**B**) Acetylated tubulin status of parental, PacR, and developed PacR phenotype cells with multiple transient cross-resistance. Cells were grown in the absence or presence 20 nM PTX for 18 hr, and the amount of acetylated tubulin was measured by immunoblotting using antibody specific to acetylated tubulin. (**C**) Mixing tubulin experiment. Indicated cells were harvested by adding hypotonic buffer with 50 μg/mL PTX for 15 min. Whole cell lysate from PacR phenotype was added to that of developed PacR/5-FU subline at different ratios as indicated, and incubated for an additional 10 min. The polymerized (Pol) and soluble (Sol) protein fractions were processed as described in Materials and Methods. (**D**) Schematic diagram of transient gene transfection and/or siRNA silencing with respective TUBB3 feedback results used in E and F in A549-PacR/5-FU cells. (**E**) Confirmation of TUBB3 feedback gene expressions after transient transfection and/or siRNA silencing of indicated genes with C as control (respective empty vector and/or scramble siRNA) and TS as the transfection scheme shown in D. (**F**) Microtubule stability of A549-PacR/5-FU cells through tubulin polymerization after indicated transient gene and/or siRNA transfections. Cells were grown in the presence of 60 nM PTX for 18 hr. The polymerized (Pol) and soluble (Sol) protein fractions were processed as in C. (**G**) Illustration of proposed mechanism behind the feedback response of TUBB3 to silencing of FOXO3a and ABCB1 in regulating microtubule stability in A549-PacR/5-FU cells.

Notably, although several cancer cell models of mt-stabilizing drug resistance (e.g., taxol) have been concomitantly associated with reduced mt stability [47, 48], a precise characterization of tubulin dynamics in A549 cells with acquired MDR has not yet been well-described. We then identified whether the tubulin dynamics in the resistant phenotypes we developed had changed relative the mt status of parental A549 cells. The tubulin microtubules (pellet fraction, P) were measured after being separating from free tubulin in the cytosol (supernatant fraction, S). The cells confirmed to have PTX resistance had lower levels of tubulin in their microtubules compared to the parental cells. This manifestation was attenuated by the subsequent acquisition of 5-FU cross-resistance. Surprisingly, although it is a PTX-analogue, transient cross-resistance to DCT strikingly destabilized tubulin dynamics, and the development of cross-resistance to a combination of 5-FU and DCT completely undermined tubulin levels in mt. In PacR cancer cells with cross-resistance to 5-FU, DCT, and 5-FU+DCT, the regulation of tubulin-dependent mt stability, which showed an impaired mt status, was evident and was strictly responsive to increasing concentrations of PTX (Figure 7A). This suggests that the acquisition of PTX resistance has less stable mt and that the development of cross-resistance to other agents can augment this event, showing more impaired mt dynamics through the modification of tubulin in mt. The stability of mt can be represented by the acetylation status of mt because post-translationally, tubulin is modified by mt acetylation, which directs conserved mt dynamics [49]. Because less tubulin was found in A549 cells with PTX-resistance and developed cross-resistance, this prompted us to measure the acetylated microtubules in the lysates of A549, A549-PacR, and A549-PacR/5-FU cells treated with or without PTX using an acetylated tubulin-specific antibody. Acquired PTX resistance and cross-resistance resulted in lower acetylation of tubulin than parental cells (Figure 7B). Combined with the reduced amount of mt found in the MDR phenotype, these data suggest that first-line PTX resistance confers less stable microtubules, which can further be augmented with the consequential onset of cross-resistance, at least to 5-FU.

To examine whether a soluble factor required for tubulin polymerization was influenced in the resistant sublines and whether it can differentiate, to a certain degree, the less stable mt we found as described above, we performed a mixing experiment. Whole cell lysates from A549-PacR cells were added to the lysate from A549-PacR/5-FU cells at varying ratios of 1:1, 1:4, and 1:19 (PacR:PacR/5-FU) to examine whether PTX resistance could affect the state of polymerization in the 5-FU cross-resistant lysate, which we further associated with a PTX-targeted mt mechanism by incorporating PTX treatment. In A549-PacR cells, although slightly attenuated, most of the tubulin is present in the polymerized form (Pol), in contrast to A549-PacR/5-FU cells, in which the majority of the tubulin is in the soluble form (Sol). This is an interesting observation because a significant difference has been identified between PacR and PacR/5-FU cells associated with tubulin polymerization. Further, the addition of lysate from PacR cells at a higher fraction of PacR/5-FU lysates did not induce polymerization of tubulin but was more polymerized when the lysate fraction from the PacR/5-FU variant was decreased (Figure 7C). Thus, there is partially an induction of polymerization amended by PacR cells to 5-FU cross-resistant subline PacR cells, which is associated with the recruitment of tubulin in the presence of polymerized tubulin. Next, we addressed the functional mechanism by which modification on *TUBB3* transcription affects mt stability and tubulin polymerization. In A549-PacR/5-FU cells, upon incorporating the transient transfection of *FOXO3a* and *ABCB1* genes or siRNAs, *TUBB3* gene expression can be modified correspondingly. Using these feedback control patterns, as verified by qRT-PCR (see scheme in Figure 7D, 7E), we subjected the control transfected cells (EV) and transfection scheme-dependent transfected cells (TS) based on the *TUBB3* response to the same assay of mt stability as that described in the method used for Figure 7C. The successful negative regulation of *TUBB3* through the co-transfection of both *FOXO3a* and *ABCB1* siRNAs conferred stabilization of the cytoskeletal mt through tubulin dynamics while preserving *TUBB3* transcription in cells transfected with *FOXO3a*-GFP and *ABCB1* siRNA. *FOXO3a* siRNA and *ABCB1*-GFP were characterized as no-feedback and did not significantly induce any changes in the undermined mt tubulin levels after the treatment with PTX (Figure 7F); similar patterns were obtained in those without PTX treatment (Supplementary Figure 9). As reported previously, disrupted *TUBB3* functioning underlies impaired mt stability whereas missense mutations causing the loss of *TUBB3* can induce increased mt stability [50, 51]. This led us to hypothesize that the development of 5-FU cross-resistance in our PacR variant might have induced mutations in *TUBB3*, thus causing its FOXO3a-directed negative regulation feedback to increase mt stability, in part by increasing polymerized tubulin (Figure 7G). These findings indicate that incidence of the cross-resistance phenomenon in PTX-resistant cancers results in less mt stability associated with tubulins and their polymerization and that the regulation of mt dynamics can be coordinated directly by *TUBB3* genetic modifications, particularly by targeting *FOXO3a*-mediated *ABCB1* activity.

## DISCUSSION

PTX and its synthetic analogues have emerged as effective and clinically validated chemotherapeutic agents targeting the mt cytoskeleton with broad activity in solid tumors and hematological malignancies [52, 53]. However, the occurrence of altered tubulin isotype expressions such as class III β-tubulin (encoded by *TUBB3*) is prominent in cancers receiving taxane-based therapy [54]. The factors contributing to genetic modifications of *TUBB3* have been poorly understood in the context of acquired drug resistance in several malignancies. Contrasting studies have been the center of controversy portraying tubulin point mutations at the PTX-binding sites conferring drug resistance. Previous reports noted disparities in the involvement of mutations in the class I β-tubulin isotype providing DNA sequencing of the fourth exon, which contains the paclitaxel-binding site, with primary drug resistance [54, 55]. This discrepancy was further supported with the results of the DNA sequence for Kα1-tubulin, in which mutations were absent in both sensitive and resistant patients with invariable SNPs independently affecting patients' responses to PTX [56, 57]. These clinical events shifted the focus to β_III_-tubulin-associated multiple genetic alterations in aggressive and drug refractory cancers because β_III_-tubulin has been mechanistically correlated with tubulin-binding agents (TBAs) although it is poorly understood in the context of drug resistance [58]. Accordingly, β_III_-tubulin has recently been classified as a biomarker for the onset of PTX-specific relapse for some cancer types. Thus, there is urgency in identifying molecular signatures that result in the modification of β_III_-tubulin at the transcriptional level in PTX-resistant cancers to accelerate the design of MDR reversal agents.

The present study demonstrates that *TUBB3* transcription can be controlled by *FOXO3a*-mediated *ABCB1* regulation and can subsequently promote the profusion of multiple cross-resistance and govern the tumor progression of PTX-resistant cancers. In a panel of normal and tumor cells that were primarily derived from the lung and prostate, we found that P-gp-associated drug-resistant (PacR and GefR) cancer cells express higher *TUBB3* and *FOXO3a* levels compared to drug-sensitive normal cells. Notably, we determined that our developed PTX-resistant cancer cells display cross-resistance to a variety of chemically different drugs including 5-FU, CIS and the PTX-analogue DCT. This occurrence led us to develop a transient cellular model of this cross-resistance. The development of transient cross-resistance to drugs that are chemically different from PTX induced regulation in *FOXO3a* and *TUBB3* endogenous expressions, encouraged escape from etoposide- and verapamil-induced cell death and was highly associated with P-gp hyperactivation. In support, we found regulated apoptotic signals and escalated intracellular ATP levels, which demonstrate a complementary mechanism by which MDR acquisition is associated with a drug response. Furthermore, drug-induced activation of *FOXO3a* up-regulates P-gp/*ABCB1* expression at gene promoter levels, which promotes cell death regulation and increases cell survival [55, 59]. We currently lack experimental evidence on the genetic drivers that specify the augmentation of chemically different acquired drug cross-resistance in tumors that developed prior to first-line acquired drug resistance, which is a well-known characteristic of MDR aggression. Here, to address this particular issue and to shed light on the onset of multiplicity of cross-resistance defining MDR, we examined the influence of *FOXO3a*-regulated *ABCB1* to *TUBB3* feedback control, as suspected from our preliminary findings. We found that transient *TUBB3* activation, through *ABCB1*, in response to the stimulation of *FOXO3a* expression, significantly contributes to the cross-resistance of the PTX-resistant cell population and consequently limits the efficacy of both agents where cancer cells have developed multiple resistance. Mechanistic studies clearly demonstrate that the observed *TUBB3* feedback control was physiologically, at least in part, amended by the ubiquitin-proteasome system engaged in the ubiquitin degradation pathway because the proteasome inhibitor up-regulated β_III_-tubulin expression. Further, we found that increased polyubiquitination of TUBB3 was correlated with *FOXO3a* silencing in 5-FU cross-resistant PacR cancer cells which reflects a strong transcriptional feedback control of *TUBB3*. The role of the ubiquitin-proteasome system in mt polymerization in cells has been previously described but remains underscored because malfunctioning of this system has been implicated in a variety of diseases [60, 61]. However, in our *in vitro* model of acquired PTX resistance and 5-FU cross-resistance, the observed systemic polyubiquitination of β_III_-tubulin appeared to support previous findings on the role of specific ubiquitin substrates essential for tumor drug resistance [62].

In assessing the extent by which *FOXO3a* regulates *ABCB1* transcription, we evaluated the methylation profiles of *ABCB1* promoter boxes deemed essential for P-gp activation and drug efflux pump hyperactivity conferring MDR. Of note, the transient overexpression of *FOXO3a* marginally promoted *ABCB1* methylation at the -50GC and -110GC boxes, which are highly relevant binding sites for *ABCB1* substrates. PTX and 5-FU-mediated drug-induced methylation was also observed as confirmed by COBRA at the CpG site of the Inr region of *ABCB1*, which showed increased DNA methylation. This finding appears to be critical in urging 5-FU cross-resistance in PTX-resistant cancers [63, 64]. Therefore, the regulatory effects of FOXO3a on P-gp control add another layer of complexity to the role of *FOXO* transcription factors in drug resistance and provide an understanding for conflicting activities in between. We identified the involvement of the PI3K/Akt pathway, a critical regulator of *FOXO* transcription factors, in this FOXO3a-directed regulation of P-gp at the protein expression level. Our results also underscore the association of these FOXO effectors, showing regulated levels of phospho-Akt and p27/Kip1 in *FOXO3a* overexpressing cells, which influence the control of P-gp and β_III_-tubulin. Although the regulatory activity of these signals in several drug-resistant models derived from colon and breast cancers has been reported [65, 66], this is the first study to reflect these associations in a MDR model stipulating 5-FU cross-resistance in PTX-resistant cancer cells. We also demonstrated a doxorubicin-induced increase in *FOXO3a* methylation at arginine residues and increased FOXO3a translocation from the nucleus to the cytoplasm, which sheds light on how *FOXO3a* regulates *ABCB1*. Interestingly, complimentary activation of MRP1 has been observed in *FOXO3a* overexpressing cells, depicting that the regulation of P-gp amended by *FOXO3a* influences MRP1 as an adjoining factor in conferring cross-resistance in PTX-resistant cancer cells.

The acquisition of PTX resistance is profoundly associated with a more aggressive and superinvasive phenotype both *in vitro* and *in vivo* [67, 68]. Although highly associated with P-gp/*ABCB1* hyperactivation, P-gp inhibitors are often ineffective and toxic at the doses required to suppress P-gp function in the clinic. In addition, PTX relapsed tumors rapidly develop MDR [69]. Our findings also indicate the contribution of secreted signals of PTX-resistant cancer cells with 5-FU cross-resistance to the outgrowth and regulation of *FOXO3a*-mediated *ABCB1* transcription, leading to a drug transporter efflux increase. Because these secretomes are unknown, we are not able to directly assess the interaction with P-gp. Despite this, we demonstrate that controlling the feedback of *TUBB3* predicts P-gp-mediated efflux function influenced by complex drug-resistant secretomes *in vitro*. Furthermore, 5-FU cross-resistant PacR cancer cells display increased EMT associated with P-gp, which can be repressed by transient silencing of *TUBB3*. Notably, the knockdown of TUBB3 resulted in the attenuation of PacR/5-FU and PacR/CIS cancer cell-induced tumors *in vivo* associated with regulated levels of Akt and VEGF. Our study identifies that the response of *TUBB3* from *FOXO3a*-regulated *ABCB1* is a critical feedback mechanism to genetically identify cross-resistance in PTX-resistant cancers and its tumor progression.

**Figure 8:**
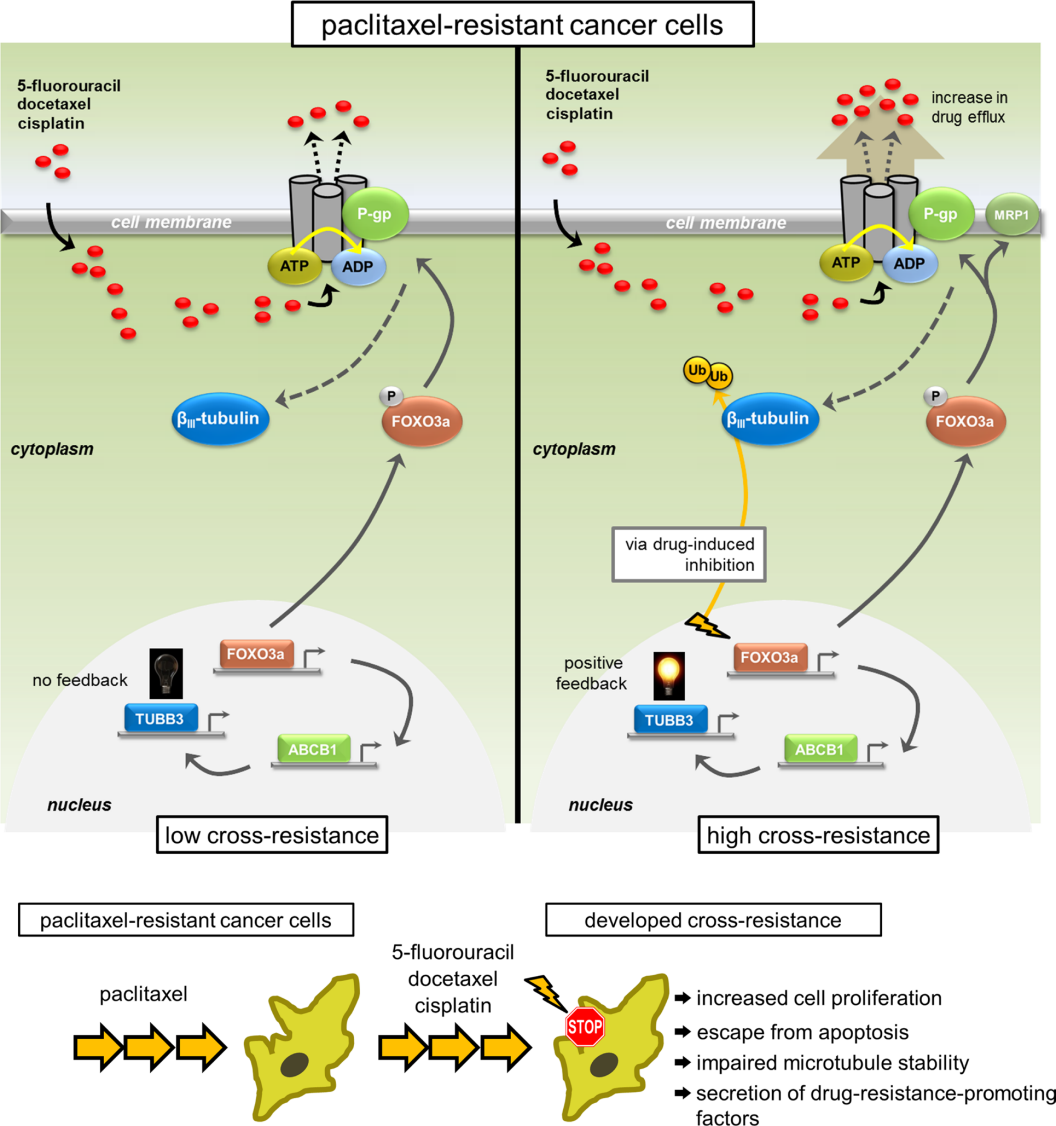
Model representation of the feedback control of TUBB3 via FOXO3a-mediated ABCB1 regulation inducing multiplicity in acquired cross-drug resistance Multiplicity of acquired cross-resistance to structurally different drugs in cancer cells selected for taxane resistance is associated with feedback control of TUBB3 through FOXO3a-mediated ABCB1 regulation inducing hyperfunctional P-gp-associated drug efflux and escape from potential inhibition.

Dysfunction in mt stability and tubulin polymerization is associated with PTX resistance in several malignancies [70]. These particular underpinnings drove us to determine the status of mt dynamics in our PTX-resistant cancer cells with a transient cross-resistance model. In particular, we found that impaired mt stability was correlated with the acquisition of the PacR, PacR/5-FU, PacR/DCT, and PacR/5-FU+DCT phenotypes. The lower acetylation level of tubulin in these models compared to the parental phenotype confirms irregularity in mt dynamics. Interestingly, we found complete attenuation of polymerized tubulin in PacR/5-FU cells compared to PacR cells with higher tubulin polymerization. Furthermore, we found that transiently silencing *TUBB3* reversed the impaired mt stability in PacR/5-FU cells. Finally, the most significant conclusion of our study is that inducing *FOXO3a* activation mediates an upsurge in *ABCB1* transcription and P-gp function and collectively controls the *TUBB3* gene response to confer multiplicity in cross-resistance to chemically different drugs in PTX-resistant cancer cells (Figure 8). Our study opens avenues for understanding how PTX-resistant cancers develop MDR and subsequently develop metastatic tumors. Our work also provides a rationale for manipulating *TUBB3*/β_III_-tubulin as a biomarker not just for PTX-resistant cancers but also for cross-resistance to a broad class of drugs.

## MATERIALS AND METHODS

### Reagents

DMEM, DMEM High Glucose, RPMI-1640 medium, M199, FBS, FCS, DPBS, antibiotic-antimycotic solution, trypsin-EDTA, TRI reagent, apoptosis kit with FITC annexin V and PI, Lipofectamine 3000 and PLUS reagents, Oligofectamine, and Opti-MEM Reduced Serum medium were purchased from Invitrogen (Grand Island, NY). siLentFect RNAi reagents were purchased from Bio-Rad (Hercules, CA). TCA reagent, MTT dye, PI, RNase A, crystal violet, sapphire 700, NAC, Rho-123 dye, BrdU, mouse anti-BrdU peroxidase, anti-tubulin, anti-acetylated tubulin, anti-actin, and HRP-conjugated secondary antibodies were purchased from Sigma-Aldrich (St. Louis, MO). Rabbit anti-β_III_-tubulin, anti-p27/Kip1, anti-vimentin, anti-VEGF, rat anti-MRP1, mouse anti-P-gp, and anti-dimethyl arginine were purchased from Abcam (Cambridge, UK). Rabbit anti-FOXO3a, anti-phospho-FOXO3a (ser318/321), anti-Akt, anti-phospho-Akt (ser473), anti-cyclin D1, anti-TCF8/ZEB1, anti-E-cadherin, anti-Snail, anti-β-catenin, and anti-myc-tag were purchased from Cell Signaling Technology (Danvers, MA). Mouse anti-phospho-ERK (Tyr204), anti-IgG, anti-c-myc, rabbit anti-IκB-α, anti-cyclin A, anti-cyclin E, and anti-Rb, were purchased from Santa Cruz Biotechnology (Santa Cruz, CA). Gene-specific primers for real-time RT- and methylation-specific PCRs and ELISA were synthesized; and gene-specific plasmids and vectors were synthesized from Origene Technologies (Rockville, MD) and Bioneer (Daejeon, Korea). The Reverse Transcription, Pgp-Glo Assay Systems, and DeadEnd Colorimetric and Fluorometric TUNEL System kits were purchased from Promega (Madison, MA). The ABCB1 PREDEASY ATPase kit was purchased from Solvo Biotechnology (Szeged, Hungary). The EnzyLight ATP Assay Kit was purchased from BioAssay Systems (Hayward, CA). Protein G sepharose beads and ECL Plus Western blotting kits were purchased from GE Healthcare (Munich, Germany).

### Compounds

Paclitaxel, 5-fluorouracil, cisplatin verapamil, and etoposide were purchased from Sigma-Aldrich. Docetaxel was purchased from LC Laboratories (Woburn, MA). MG132 was purchased from Calbiochem (San Diego, CA). Doxorubicin, gefitinib, and LY294002 were purchased from Selleck Chemicals (Houston, TX). All drugs used have > 99% purity identified through high-performance liquid chromatography and made commercially available.

### Cell culture

The human normal prostate (RWPE-1), normal lung (L132, MRC-5), prostate cancer (PC-3), and lung cancer (H292) cell lines were obtained from the Korean Cell Line Bank (Seoul, Korea). The human lung cancer (A549), embryonic kidney (HEK293), and umbilical vein endothelial (HUVEC) cell lines were obtained from the American Type Culture Collection (Manassas, VA). Every 6 months, all cell lines were tested for mycoplasma contamination using the mycoplasma PCR detection kit (Intron Biotechnology, Sungnam, Korea). Cells were grown in medium (K-SFM for RWPE-1; DMEM for L132, MRC-5, and HEK293; RPMI-1640 for A549, PC-3, and H292 cells) supplemented with 10% FBS and antibiotics-antimycotics (100 units/mL penicillin G sodium, 100 mg/ml streptomycin, and 250 ng/ml amphotericin B [PSF]). HUVEC cells were grown in M199 medium with 20% HI-FBS, 20 μg/ml ECGS, and 50 μg/mL heparin. All cells were cultured at 37°C and 5% CO_2_ in a humidified atmosphere.

### Establishment of drug resistant cell lines and transient cross-resistant phenotype

Paclitaxel-resistant A549 (A549-PacR), PC-3 (PC-3-PacR), and gefitinib-resistant H292 (H292-GefR) cells were originally developed by our team [71, 72] derived from their respective parental phenotype through continuous exposure to gradually increasing concentrations of the drug for > 12 months maintaining continuous growth and fine parental-like morphology. Drug resistant cells were maintained in medium containing low-dose drug and were found to have > 200 resistance fold. PTX-resistant (A549- and PC-3-PacR) cells were developed to have transient cross-resistance to 5-fluorouracil (PacR/5-FU), docetaxel (PacR/DCT), and cisplatin (PacR/CIS) by culturing PTX-resistant cells in serum-rich (20–25% FBS) medium for 3 days followed by exposure to gradually increasing concentrations of structurally-different drugs for 12 days (5-FU, 2.5–75 μM; DCT, 10–60 nM; CIS, 2.5–50 μM) reaching a high-dose were cross-resistant cells survived and were selected in a serum-starving medium (20%, 10%, 5%, 2%, 0% FBS intervals following subculture). After cross-resistance selection, cells were exposed to low tolerating dose of drugs (5-FU, 5–30 μM; DCT, 25–60 nM; CIS, 5–25 μM) for 20 days in a serum-enriching medium (0%, 5%, 10%, 20% FBS intervals following subculture). Cells were subcultured for every 48-56 hr cycle. Apoptotic cell morphology and P-gp and MRP1 MDR-marker protein expression check-ups were conducted at days 7 and 19 of the > 35 days of transient cross-resistant phenotype development (Supplementary Figure 6). PTX-resistant cells with confirmed cross-resistance were maintained in medium containing low-dose drug (1/25–1/30 of their respective IC_50_s). All drug-resistant subline cells were cultured in medium in the same formulation and condition as with their parental phenotype.

### RNA extraction, real-time RT-PCR, methylation-specific PCR, and COBRA

RNA extraction and real-time RT-PCR were performed as described previously [71]. Genomic DNA isolation, bisulphite treatment of DNA, and methylation-specific PCR were carried out as described previously [73, 74]. Bisulphite DNA modification, primer design for and combined bisulphate restriction analysis (COBRA) of ABCB1 were performed as described previously [74]. All primer sequences are listed in Supplementary Table 1.

### Western blot analysis and immunoprecipitation

Preparation of lysates, protein quantification, SDS-PAGE, and Western blot analysis were carried out as described previously [76]. Immunoprecipitation was performed as described previously [76].

### Plasmids, gene transient transfections, and RNAi

For ABCB1 overexpression studies, cells were dual transfected with pCMV6-AC-GFP (empty vector) and pCMV6-AC-ABCB1 (GFP-tagged ABCB1) using Lipofectamine 3000 (Life Technologies). The wild type ABCB1 expression vector consists of full-length ABCB1 (MDR1) cDNA within the pCMV6 mammalian expression vector (Origene Technologies). For FOXO3a overexpression studies, cells were dual transfected with pEGFP-N1 (empty vector) and pEGFP-N1-FOXO3a using Lipofectamine 3000. The fusion GFP-tagged FOXO3a-WT encoding vector was constructed by subcloning the full-length FOXO3a cDNA (Clontech, Palo Alto, CA). Gene-specific methods used for transient transfections were described previously [78, 79]. For ABCB1 silencing, cells were transfected with scramble siRNA (FITC-conjugate) or human-specific ABCB1 (MDR1) siRNA duplexes (Santa Cruz Biotechnology) using Lipofectamine 3000 [80]. For FOXO3a silencing, cells were transfected with stealth scramble or FOXO3a siRNAs (Santa Cruz Biotechnology) using Lipofectamine 3000 [81]. For TUBB3 silencing, cells were transfected with scramble or TUBB3 siRNA oligos (Ambion, Austin, TX) using siLentFect (Bio-Rad) [82]. Transfectants were subjected to drug treatment and/or assays after 48 hr of transfection. RNAi and transient gene transfection efficiencies and cell morphology are presented in Supplementary Figure 7.

### Immunocytochemistry

Immunofluorescences of cells were observed using Zeiss LSM 780 ApoTome microscope (Carl Zeiss, Jena, Germany). Fluorescence imaging and immunocytochemistry were performed as previously described [71].

### Cell proliferation and colony formation assays

MTT assay was carried out to determine cell proliferation as described previously [73]. Colony formation assay using crystal violet or sapphire 700 staining was performed as described previously [83].

### Apoptosis, BrdU incorporation, and cell cycle assays

Assessment of apoptotic cells using Annexin-V-FITC and PI double staining kit was performed as described previously [84]. BrdU incorporation assay and cell cycle analysis were performed as described previously [71]. All fluorescent events were acquired using a FACS-Calibur equipped with CellQuest Pro software (BD Biosciences, San Jose, CA).

### ABCB1 and P-gp atpase activity, Rho-123 assay, and ATP quantification

ATPase activity determination was carried out using ABCB1 PREDEASY ATPase kit and Pgp-Glo Assay System kit according to manufacturer's instructions (Promega and Solvo Biotechnology, respectively). Rho-123 accumulation assay was carried out as described previously [71]. Determination of intracellular ATP content was performed using EnzyLight ATP Assay kit according to manufacturer's instructions (BioAssay Systems).

### Isolation of cytosolic and nuclear extracts

Cells were harvested at 80% confluence through trypsination. Isolation of nuclei and cytosol was carried out using NE-PER Nuclear and Cytoplasmic Extraction Reagents (Pierce, Rockford, IL) following manufacturer's instructions.

### Cell migration, invasion, and soft agar assays

Chemotaxis-induced migration and cell invasion assays were carried out in Boyden chamber wells with or without matrigel-based membrane as described previously [73]. Anchorage-independent growth was assessed in a two-layer agar system with final concentration of 0.8% for the bottom and 0.4% for the top agarose layers according to manufacturer's protocol (Millipore, Bedford, MA).

### *In vivo* tumor xenograft model, tumor immunohistochemistry, and *ex vivo* biochemical analysis

All animal use and care followed the ethical guidelines of and approved by the Seoul National University Institutional Animal Care and Use Committee. 5–6 weeks old male (BALB/c-nu) athymic mice were purchased from Hyochang Science (Daegu, Korea). Viable cells were determined by trypan blue exclusion test and a total cell density of 3 ×10^7^ from each group (*n* = 2 to 4) were collected. Bulk cell suspensions were diluted in matrigel (1:1 mix ratio) and were injected subcutaneously into the upper and lower right and left flanks. All mice did not receive any drug treatment after injection. All mice were mercifully killed at day 35 and the tumor were removed, scaled, and subjected to further analysis. Immunohistochemistry of tumors, H&E staining, and *ex vivo* biochemical analysis were performed as described previously [77]. TUNEL assay of paraffin-embedded tissues were carried out according to manufacturer's protocol using the DeadEnd TUNEL System kits (Promega).

### Microtubule stability assessment, tubulin polymerization and acetylated tubulin measurement

Mt stability was assessed with slight modification as described previously [85]. Degree of tubulin polymerization, mixing experiment, and measurement of relative acetylated tubulin in cells were carried out as described previously [4].

### Software, statistics, and image acquisition

Statistical analyses were assessed (*p* < 0.05) and performed using OriginPro 8.0 (OriginLab, Northampton, MA). IC_50_ values of all compounds and non-linear regression were calculated using the TableCurve 2D v5.01 (Systat Software Inc., San Jose, CA). Statistical evaluation were carried out using one-/two-way ANOVA with Bonferroni's multiple comparisons post -test and Student's *t* test and *x*^2^ test. GraphPad Prism 5.01 software was used to calculate the mean fluorescence intensity (MFI). Adobe Photoshop software was also used for image acquisition aid and only brightness and/or contrast of expression blots were simultaneously changed for all areas only if deemed necessary. All results were obtained from at least three independent experiments and most were biologically replicated. The data are presented as mean ± SD.

## SUPPLEMENTARY MATERIALS FIGURES AND TABLE


