# Supplementary material for: Multiplicity of acquired cross-resistance in paclitaxel-resistant cancer cells is associated with feedback control of TUBB3 via FOXO3a-mediated ABCB1 regulation
Source: Oncotarget. 2016 Apr 30;7(23):34395–419. doi: 10.18632/oncotarget.9118 (PMC5085164; doi:10.18632/oncotarget.9118)
Supplement: Supplementary file 1 [file oncotarget-07-34395-s001.pdf]

Multiplicity of acquired cross-resistance in paclitaxel-resistant cancer cells is associated with feedback control of TUBB3 via FOXO3a-mediated ABCB1 regulation

Supplementary Materials

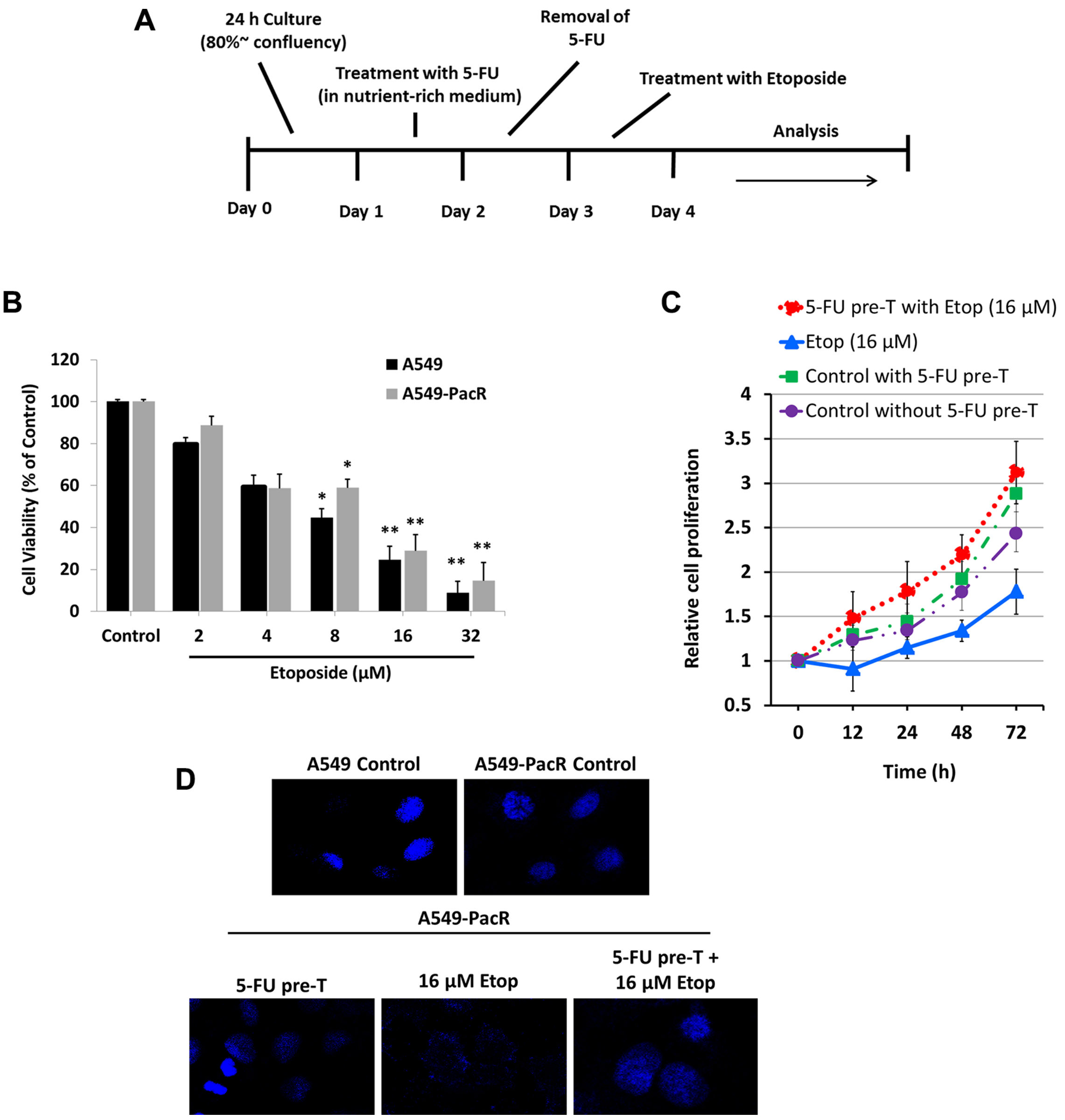

Supplementary Figure S1: Extended 5-FU treatment supports escape from etoposide-induced apoptosis in PTX-resistant A549 cells. (**A**) Scheme of drug treatments in A549-PacR cells used for further analysis in C and D. (**B**) Cell viability of A549 and A549-PacR cells after 18 hr exposure to various concentrations of etoposide. Cell viability was determined by MTT assay. Data are represented as means ± SEM. (**C**) Assessment of A549-PacR cell proliferation following drug-treatment scheme in A. Cell viability was determined by MTT assay. Data are represented as means ± SEM. (**D**) Nuclear morphology of A549 and A549-PacR cells with or without receiving etoposide and 5-FU treatment for 24 hr. Combination of 5-FU and etoposide is described in B. Cells were stained with DAPI and analyzed by confocal microscopy.

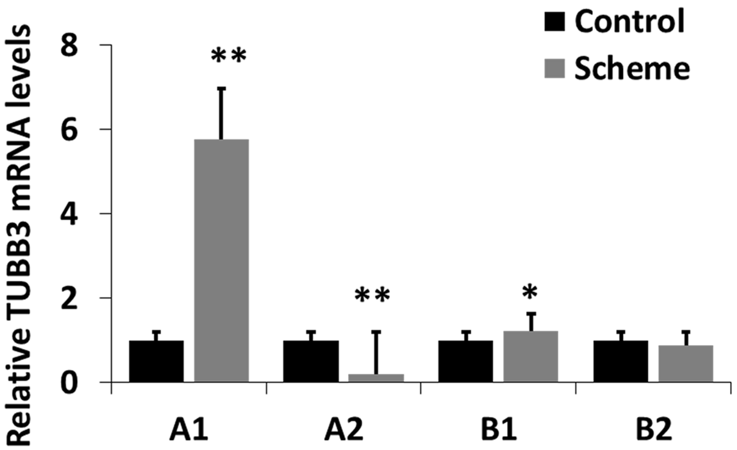

Supplementary Figure S2: *TUBB3* expression gene response to *FOXO3a* and *ABCB1* modifications in cross-resistant cell secretome-stimulated PTX-resistant cells. TUBB3 gene levels after the transient knock-out and/or knock-in of FOXO3a and ABCB1 genes. Gene expression levels were analyzed by qRT-PCR. Indicated controls are those cells that received empty vector and/or scramble siRNA. Schematic procedure is based on what is shown in Figure 5K.

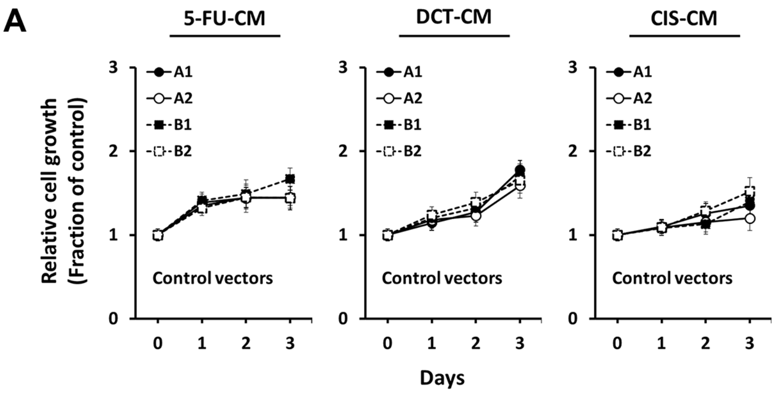

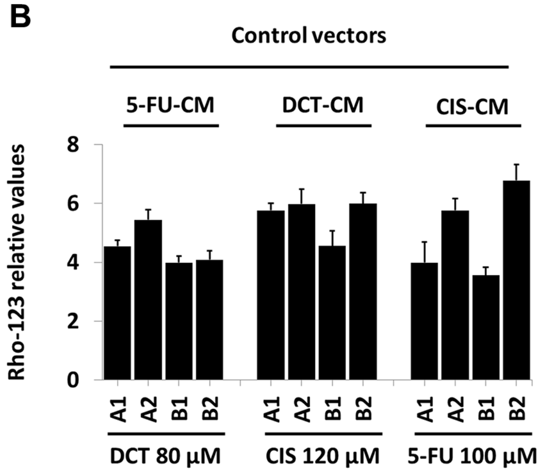

Supplementary Figure S3: Cell growth and P-gp drug efflux activity in control cross-resistant PacR cells with modified *FOXO3a* and *ABCB1* levels in response to drug treatment. (**A**) Cell proliferation of A549-PacR cells transfected with the control vectors (empty vectors or scramble siRNA) in response to drug-specific stimulated CM in the same procedure as shown in schematic A of Supplemental Figure 2. Cell viability was analyzed by MTT assay. Data are represented as means ± SEM. (**B**) Rhodamine-123 dye accumulation in A549-PacR cells transfected with the control vectors (empty vectors or scramble siRNA) in response to drug-specific stimulated CM in the same procedure as shown in schematic K of Figure 5. Cells were then treated with indicated DCT, CIS, and 5-FU concentrations for 24 hr. Drug efflux was then analyzed by flow cytometry. Data are represented as means ± SEM.

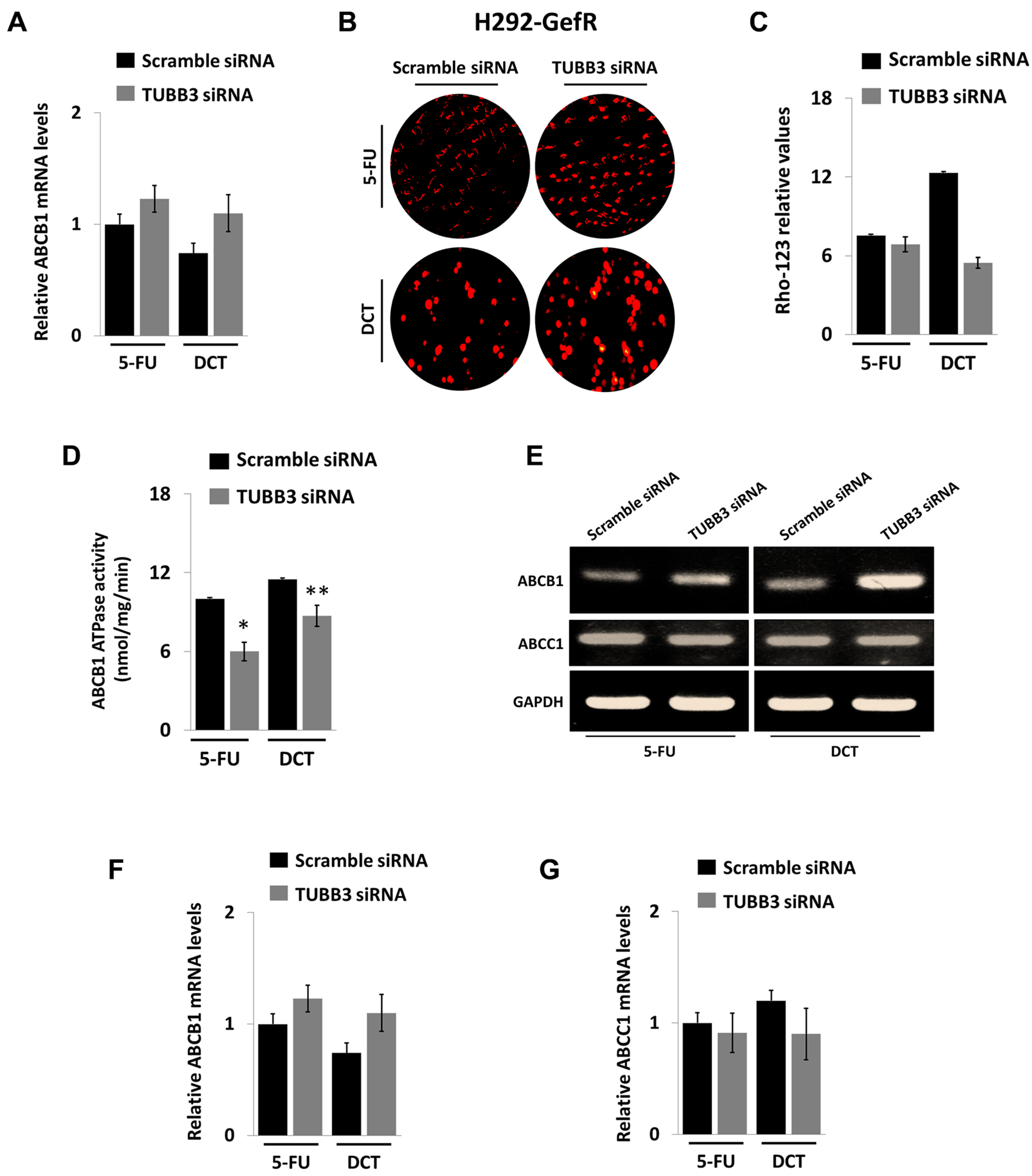

Supplementary Figure S4: Transient knock down of *TUBB3* does not significantly affect P-gp function in GEF-resistant H292 cells. (**A**) Fraction of drug resistant H292-GefR cells transfected with either scramble or TUBB3 siRNA for 48 hr, in response to 24 hr-treatment with 5-FU or DCT. Cell viability was assessed by MTT assay. Data are represented as means ± SEM. (**B**) Colonies of H292-GefR cells transfected with either scramble or TUBB3 siRNA for 48 hr, in response to low-dose treatment with 5-FU or DCT. Colonies were stained with sapphire 700. (**C**) Rhodamine-123 dye accumulation in H292-GefR cells with same transfection and drug treatment procedure as in A. Rho-123 was measured by flow cytometry. Data are represented as means ± SEM. (**D**) ABCB1 ATPase activity of H292-GefR cells after the same scheme as in A. Cells were analyzed by flow cytometry. Data are represented as means ± SEM. (**E**) Gene expression transcripts of ABCB1 and ABCC1 after the same scheme as in A. Gene levels were analyzed by RT-PCR. (**F** and **G**) ABCB1 (F) and ABCC1 (G) gene levels were measured after the scheme in A by qRT-PCR. Data are represented as means ± SEM.

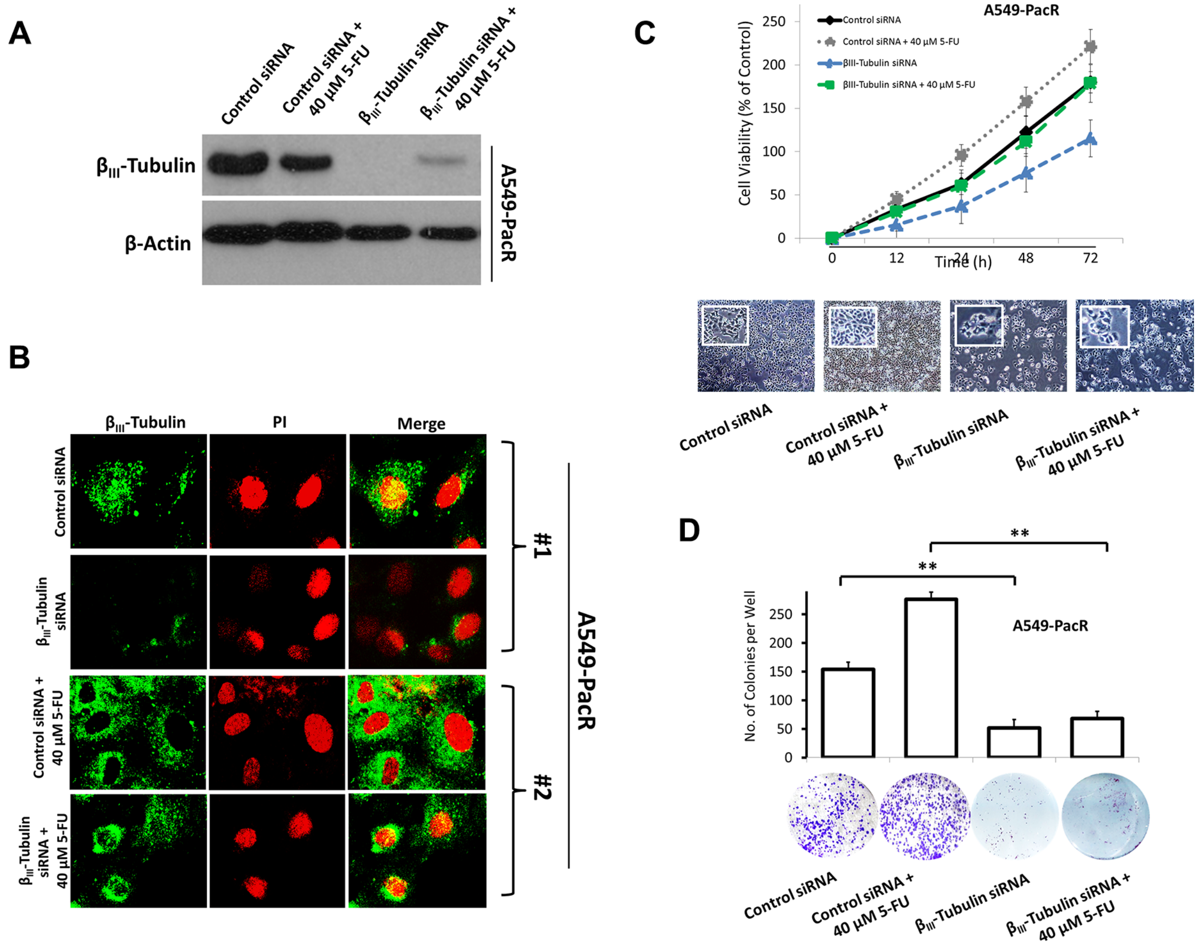

Supplementary Figure S5: Transient silencing of TUBB3 prevents 5-FU stimulated outgrowth of PTX-resistant A549 cells. (**A**) Protein expression of β_{III}-tubulin after transfection with either scramble or TUBB3 siRNA in combination with or without 40 µM of 5-FU for 24 hr in A549-PacR cells stimulated with 5-FU as in the same scheme in Supplemental Figure 1B. Whole cell lysates were analyzed by Western blotting. (**B**) Intracellular distribution of β_{III}-tubulin after indicated transfection and 5-FU treatment. Cells were stimulated with low dose 5-FU before subjecting to transfection. Cells were stained with PI (nuclei) and β_{III}-tubulin antibody followed by AlexaFluor 488 (green). Images were taken using confocal microscope. Notice that non-treated and 5-FU-treated groups were of different classification- two groups were biologically conducted separately (#1, #2). (**C**) Cell proliferation overtime of A549-PacR cells tubulin after transfection with either scramble or TUBB3 siRNA in combination with or without 40 µM of 5-FU for 24 hr in A549-PacR cells stimulated with 5-FU as in the same scheme in Supplemental Figure 1B. Cell viability was assessed by MTT assay. Data are represented as means ± SEM. (**D**) Cellular morphology of A549-PacR cells after the same transfection and treatment procedures as in C. Images were taken using phase-contrast microscope. (**E**) Colony formation of A549-PacR cells stimulated with low-dose 5-FU after the same transfection and treatment procedures as in C. Colonies were stained with crystal violet. Formed colonies were counted using the ImageJ NIH software. Data are represented as means ± SEM.

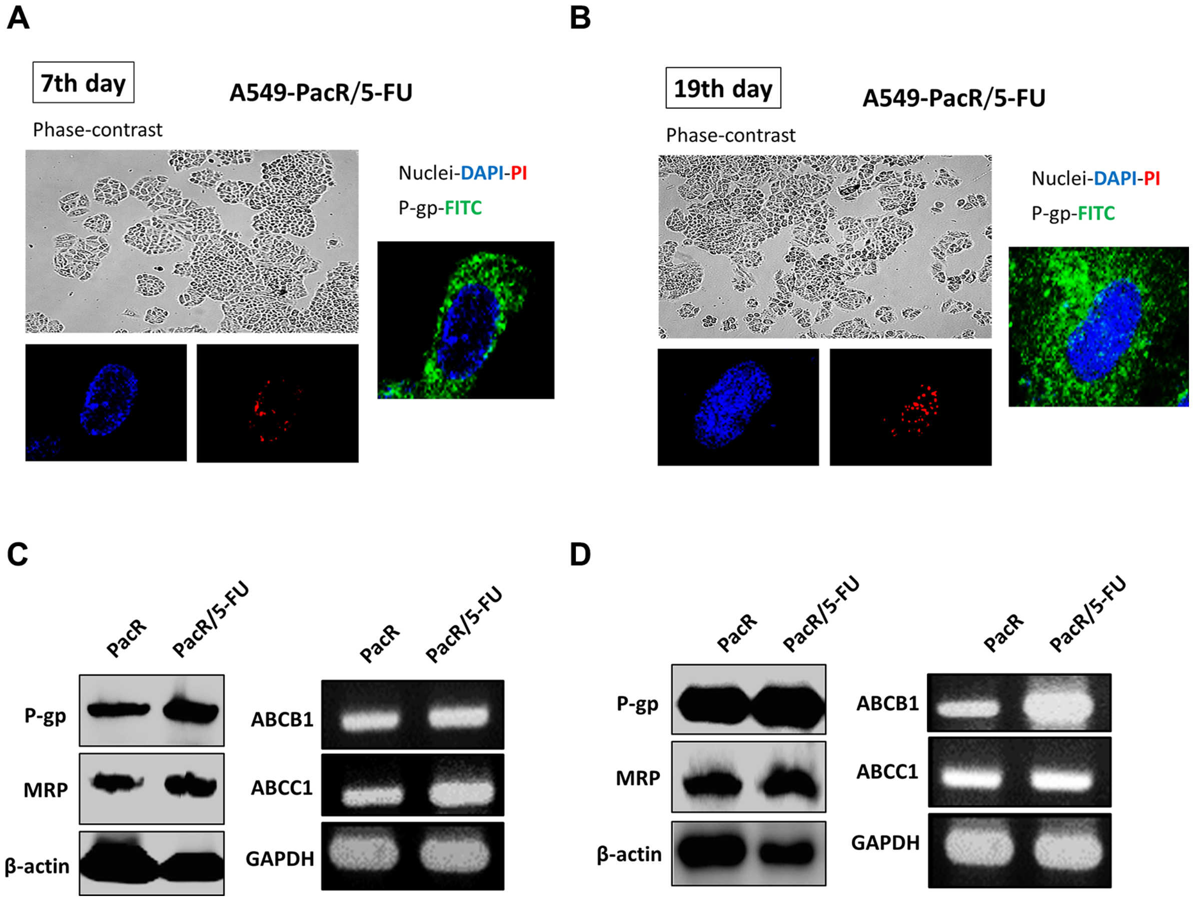

Supplementary Figure S6: P-gp-associated acquired drug cross-resistance analysis days 7 and 19 of the transient establishment of 5-FU cross-resistant A549-PacR cells. (**A** and **B**) Cellular morphology and intracellular distribution of P-gp at days 7 (A) and 19 (C) of the transient establishment of 5-FU cross-resistance in A549-PacR cells. Cells were stained with DAPI or PI for nuclei and FITC conjugated P-gp antibody for P-gp detection. Cells were analyzed by phase-contrast microscope and confocal microscopy. (**C** and **D**) Western blot and PCR gene transcripts of P-gp (ABCB1) and MRP (ABCC1) at days 7 (C) and 19 (D) during the 5-FU cross-resistance development in A549-PacR cells. Protein expressions were detected by Western blotting and gene expressions by RT-PCR.

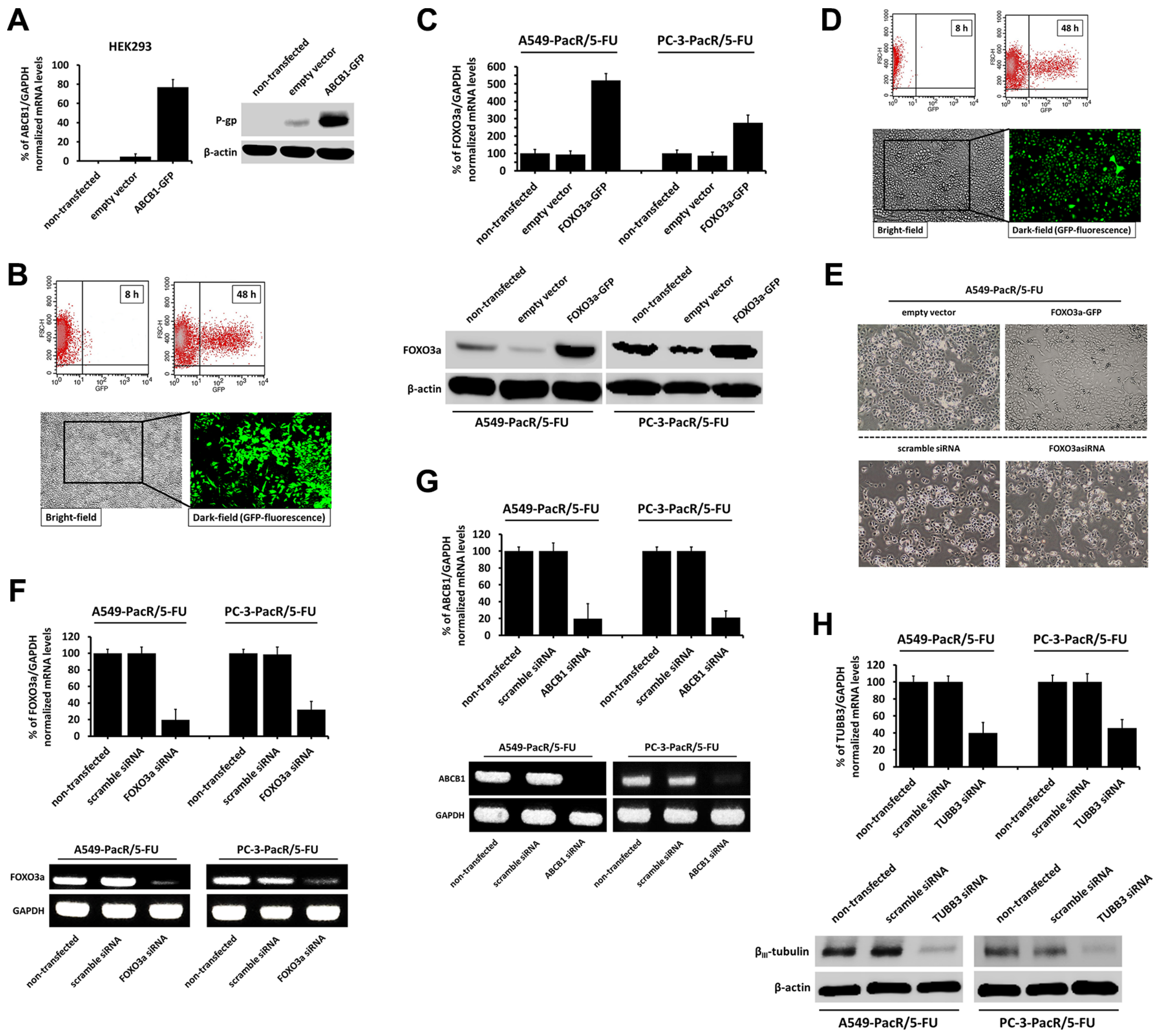

Supplementary Figure S7: Transient transfection efficiencies in various cell models. (**A**) Characterization of HEK293 cells for ABCB1 expression after transfection with or without empty vector or ABCB1-GFP. (Left panel) ABCB1 gene levels quantified by qRT-PCR and (Right panel) protein expression of P-gp through Western blotting. Data are represented as means ± SEM. (**B**) Fraction of GFP-tagged HEK293 cells after transfection with ABCB1-GFP for 48 hr analyzed by FACS (Upper panel). Cellular morphology and GFP-fluorescence of HEK293 cells after transfection with ABCB1-GFP vector (Lower panel). Images were taken through phase-contrast microscope and fluorescence microscope. (**C**) Characterization of A549-PacR/5-FU and PC-3-PacR/5-FU cells after transfection with or without empty and FOXO3a-GFP vectors for 48 hr. (Upper panel) FOXO3a gene levels after indicated gene transfections, normalized by GAPDH, and analyzed by qRT-PCR. Data are represented as means ± SEM. (Lower panel) FOXO3a protein expression after gene transfection. Lysates were subjected to Western blotting. (**D**) Fraction of GFP-tagged A549-PacR/5-FU cells after transfection with ABCB1-GFP for 48 hr analyzed by FACS (Upper panel). Cellular morphology and GFP-fluorescence of A549-PacR/5-FU cells after transfection with ABCB1-GFP vector (Lower panel). Images were taken using phase-contrast microscope and fluorescence microscope. (**E**) Morphology of A549PacR/5-FU cells after transfection with control vectors, FOXO3a-GFP, or FOXO3a-siRNA for 48 hr in a low-serum environment (0.5~2% FBS) to induce EMT. Images were taken using phase-contrast microscope. (**F**) Characterization of A549-PacR/5-FU and PC-3-PacR/5-FU cells for FOXO3a expression after transfection with or without scramble siRNA or FOXO3a-siRNA. (Upper panel) FOXO3a gene levels after indicated siRNA transfections for 48 hr. Gene levels were normalized by GAPDH and analyzed by qRT-PCR. Data are represented as means ± SEM. (Lower panel) FOXO3a gene transcripts after indicated siRNA transfections for 48 hr analyzed by RT-PCR. (**G**) Characterization of A549-PacR/5-FU and PC-3-PacR/5-FU for ABCB1 expression after transient transfection with or without scramble or ABCB1-siRNAs for 48 hr. (Upper panel) ABCB1 gene levels analyzed by qRT-PCR after indicated siRNA transfections for 48 hr. Data are represented as means ± SEM. (Lower panel) ABCB1 gene transcripts analyzed by RT-PCR after indicated siRNA transfections for 48 hr. (**H**) Characterization of A549-PacR/5-FU and PC-3-PacR/5-FU cells for TUBB3 expressions after transient transfections with or without scramble or TUBB3 siRNAs for 48 hr. (Upper panel) TUBB3 gene levels analyzed by qRT-PCR after indicated siRNA transfections for 48 hr. Data are represented as means ± SEM. (Lower panel) TUBB3 gene transcripts analyzed by RT-PCR after indicated siRNA transfections for 48 hr.

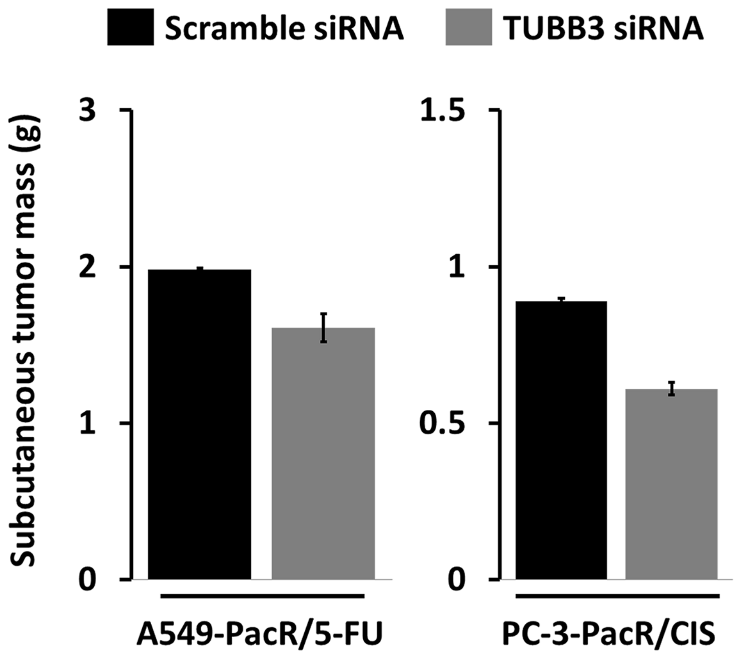

Supplementary Figure S8: Effect of *TUBB3* deficiency in drug resistant tumor growth. Subcutaneous primary tumor mass (shown in grams) induced by indicated siRNA transfected cells was measured. Data are represented as means ± SEM.

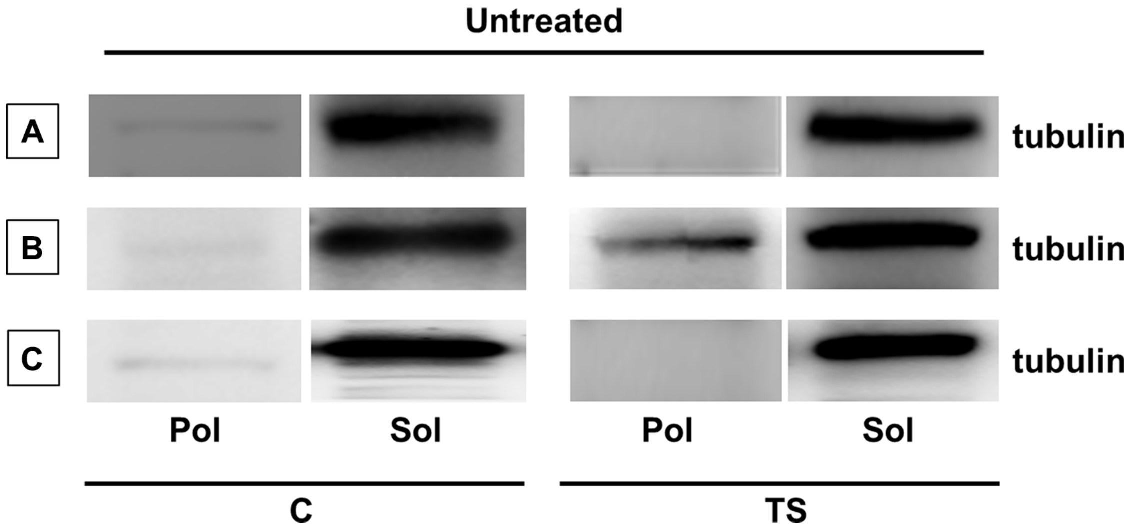

Supplementary Figure S9: Microtubule stability of A549-PacR/5-FU cells through tubulin polymerization after indicated transient gene and/or siRNA transfections. Cells were grown without the presence of PTX for 18 hr. The polymerized (Pol) and soluble (Sol) protein fractions were processed as described in Materials and Methods.

Supplementary Table S1: Primer sequences used in quantitative real-time PCR (qRT-PCR), ELISA, and methylation specific PCR (MSP)

Gene	Assay	Forward Sequence	Reverse Sequence
TUBB3	qRT-PCR, ELISA	TCAGCGTCTACTACAACGAGGC	GCCTGAAGAGATGTCCAAAGGC
FOXO3a	qRT-PCR, ELISA	TTCAAGGATAAGGGCGACAG	CAGGTCGTCCATGAGGTTTT
ABCB1	qRT-PCR, ELISA	CCCATCATTGCAATAGCAGG	GTTCAAACTTCTGCTCCTGA
ABCC1	qRT-PCR	CCGTGTACTCCAACGCTGACAT	ATGCTGTGCGTGACCAAGATCC
AKT1	qRT-PCR	TGGACTACCTGCAACTCG-GAGAA	GTGCCGCAAAAGGTCTTCATGG
VEGF	qRT-PCR	TTGCCTTGCTGCTCTACCTCCA	GATGGCAGTAGCTGCGCTGATA
GAPDH	qRT-PCR, ELISA	ATCCCATCACCATCTTCCAG	CCATCACGCCACAGTTTCC
ABCB1 promoter	ELISA, MSP	CGATCCGCCTAAGAACAAAG	AGCACAAATTGAAGGAAGGAG
